# Modification of Gene Expression, Proliferation, and Function of OP9 Stroma Cells by Bcr-Abl-Expressing Leukemia Cells

**DOI:** 10.1371/journal.pone.0134026

**Published:** 2015-07-28

**Authors:** Emmanuelle Supper, Suhail Tahir, Takahiko Imai, Joe Inoue, Nagahiro Minato

**Affiliations:** Department of Immunology and Cell Biology, Graduate School of Medicine, Kyoto University, Kyoto, Japan; B.C. Cancer Agency, CANADA

## Abstract

Expression of the *Bcr-Abl* fusion gene in hematopoietic progenitor cells (HPCs) results in the development of chronic myelogenous leukemia (CML), for which hematopoietic microenvironment plays an important role. We investigated the specific effects of an HPC line transduced with *Bcr-Abl*, KOBA, on BM-derived OP9 stroma cells. DNA microarray analysis revealed that OP9 cells co-cultured with KOBA cells (OP9/L) show diverse changes in the gene expression. OP9/L cells showed significant down-regulation of *Cdkn* genes and up-regulation of *Icam1*, leading to the increased proliferation capacity of OP9 cells and enhanced transmigration of leukemia cells through them. The effects were attributed to direct Notch activation of OP9 cells by KOBA cells. OP9/L cells also showed a markedly altered cytokine gene expression pattern, including a robust increase in a variety of proinflammatory genes and a decrease in hematopoietic cytokines such as *Cxcl12*, *Scf*, and *Angpt1*. Consequently, OP9/L cells promoted the proliferation of KOBA cells more efficiently than parental OP9 cells, whereas the activity supporting normal myelopoiesis was attenuated. In mice bearing KOBA leukemia, the characteristic genetic changes observed in OP9/L cells were reflected differentially in the endothelial cells (ECs) and mesenchymal stroma cells (MCs) of the BM. The ECs were markedly increased with Notch-target gene activation and decreased *Cdkn* expression, whereas the MCs showed a marked increase in proinflammatory gene expression and a profound decrease in hematopoietic genes. Human CML cell lines also induced essentially similar genetic changes in OP9 cells. Our results suggest that CML cells remodel the hematopoietic microenvironment by changing the gene expression patterns differentially in ECs and MCs of BM.

## Introduction

The hematopoietic microenvironment plays crucial roles in normal hematopoiesis [1, 2]. The stroma cells in the hematopoietic microenvironment represent diverse nonhematopoietic cell lineages, including mesenchymal stem and progenitor cells, osteoblasts, adipocytes, neuronal cells, and endothelial cells [3]. The bone marrow (BM) is highly vascular and features a sinusoidal structure of endothelial cells (ECs), with mesenchymal stroma cells (MCs) being located in perivascular regions forming a network between hematopoietic cell islands [4]. The stroma cells regulate hematopoiesis via direct interactions with hematopoietic cells and secretion of various hematopoietic cytokines [5, 6]. Accumulating evidence indicates that the stroma cells also affect the growth and spread of leukemia cells arising in the hematopoietic microenvironment [7–9].

Chronic myelogenous leukemia (CML) is caused by chromosomal translocations leading to the generation of *Bcr-Abl* fusion genes. CML stem cells are enriched in the same fraction as normal hematopoietic stem cells (HSCs) [10–12], and the developmental hierarchy of CML cells is analogous to that of normal hematopoiesis [13–15]. However, the proliferation and differentiation of CML stem/progenitor cells overwhelm normal hematopoiesis, resulting in the marked accumulation of myeloid progenitors and mature granulocytes. Recent reports suggest that the CML stem/progenitor cells are regulated by the microenvironment differently from normal HSCs/hematopoietic progenitor cells (HPCs) [9, 16].

The Rap1 G protein signal plays an important role in cell-cell and cell-matrix interactions [17]. We previously reported that *Bcr-Abl* strongly activates Rap1 in CML cells [18], and deficiency of *Sipa1*, a negative Rap1 regulator, enhances the sustenance of CML stem/progenitors in vivo [19]. Although the majority of *Sipa-1* ^*–/–*^mice develop CML-like myeloproliferative disease, it occurs only after long latent periods over a year, suggesting the requirement of secondary oncogenic hits [20]. It is suggested that *Sipa1*-deficiency in HPCs predisposes the overt leukemia genesis by oncogenes including *Bcr-Abl*.

In the current study, we established a nonleukemic *Sipa-1* ^*–/–*^HPC line, KOP1, whose survival is dependent on OP9 stroma cells representing authentic mesenchymal stem cells [21]. Taking advantage of the strict stroma cell-dependency of KOP1 cells, we investigated the effects of *Bcr-Abl* expression in KOP1 cells on the interaction with OP9 cells. We demonstrate that the KOP1 cells expressing *Bcr-Abl*, *KOBA*, induce diverse changes in the gene expression of OP9 cells through direct interaction, partly via Notch activation, and the effects are reflected differentially in the primary ECs and MCs of BM invaded by the KOBA cells in vivo. We further indicate that human CML cell lines induce essentially the same effects on OP9 cells. Our results suggest that CML cells remodel the hematopoietic microenvironment by directly changing the gene expression patterns of distinct types of BM stroma cells.

## Results

### *Bcr-Abl*–expressing leukemia cells enhance the proliferation capacity of OP9 stroma cells

**Fig 1:**
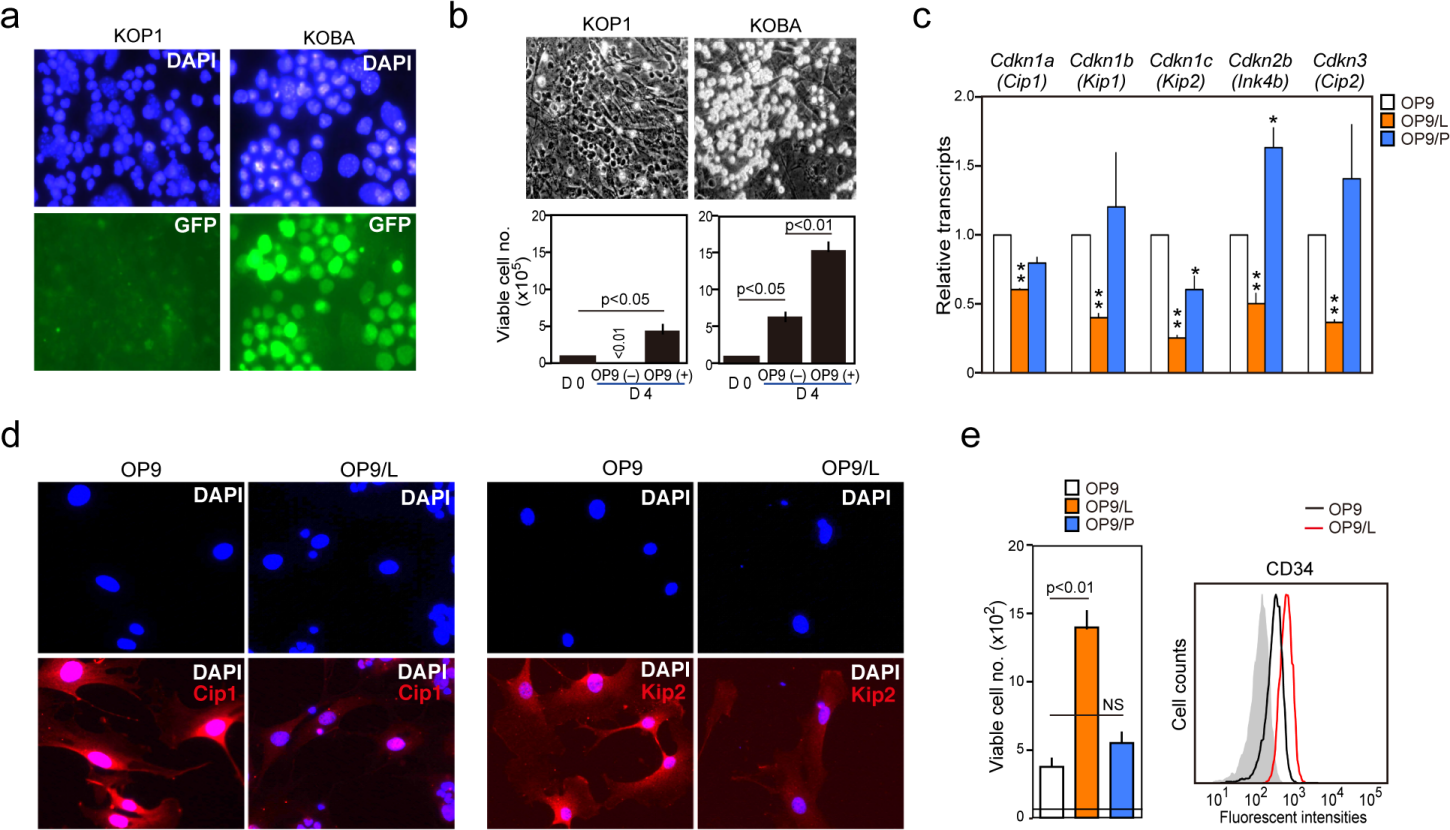
*Bcr-Abl* ^*+*^ KOBA leukemia cells repress the expression of Cdk inhibitors and enhance the proliferation of OP9 stroma cells. (**a**) KOP1 and KOBA were immunostained with anti-GFP antibody and DAPI. (**b**) KOP1 and KOBA cells (1 × 10^5^ cells) were co-cultured with OP9 cells. KOP1 cells crawled under OP9 cells, forming a cobblestone pattern, whereas KOBA cells were on top of them. Viable cell numbers of KOP1 and KOBA cells in the absence or presence of OP9 cells on day 4 are also shown. The means and standard errors (SEs) of triplicate culture. (**c**) Expression of indicated genes was determined in OP9, OP9/L, and OP9/P cells using quantitative RT-PCR. The means and SEs of triplicate determination. *P < 0.05 and **P < 0.01. (**d**) OP9 and OP9/L cells were immunostained with anti-Cip1 and anti-Kip2 along with DAPI. (**e**) OP9, OP9/L, and OP9/P cells (100 cells) were cultured alone, and the viable cell numbers were counted on day 8. NS, not significant.

Culture of the BM cells from young *Sipa1* ^−/−^ mice reproducibly resulted in the continuous cell lines that could be propagated in the presence of BM-derived OP9 stroma cells. These cells, represented by KOP1, showed immature hematopoietic cell morphology and phenotypes (Mac1^−^ Gr-1^−^ CD3^−^ B220^−/low^ c-Kit^+^ CD34^−^ CD43^+^ BP-1^+^), probably committed to a B-lineage (S1 Fig). The survival was absolutely dependent on OP9 cells and was not supported by any hematopoietic cytokines (S1 Fig). KOP1 cells showed no leukemogenic activity in normal mice even after the inoculation of 10^7^ cells per mouse (S2 Fig). Taking advantage of the absolute stroma-dependency, we intended to investigate the effects of *Bcr-Abl* expression in KOP1 cells on the interaction with OP9 stroma cells. We retrovirally transduced *p210-Bcr-Abl* in KOP1 cells (Fig 1A); as expected, the *Bcr-Abl* ^+^ KOP1 cells, termed KOBA, showed potent leukemogenic activity, killing normal mice in 3 weeks with as few as 10^4^ cells per mouse (S2 Fig). Although KOP1 cells crept underneath OP9 cells forming cobblestones in the co-culture, the majority of KOBA cells attached but stayed on top surface of OP9 cells (Fig 1B). The survival of KOBA cells was no longer OP9-dependent, although the proliferation was significantly enhanced in the presence of OP9 cells (Fig 1B).

We co-cultured OP9 and KOBA cells for 8 days, recovered the OP9 cells by depleting KOBA cells (OP9/L), and performed a comparative DNA microarray analysis with untreated OP9 cells; contamination of KOBA cells was less than 1%. The OP9/L cells showed remarkable changes in the gene expression compared to control OP9 cells (S1 Table). Among them, we noticed significantly decreased expression of a series of Cdk inhibitor genes, including *Cdkn1a* (*Cip1*), *Cdkn1b* (*Kip1*), *Cdkn1c* (*Kip2*), *Cdkn2b* (*Ink4b*), and *Cdkn3* (*Cip2*). The effects were specific for KOBA cells and were barely detected in the OP9 cells co-cultured with parental KOP1 cells (OP9/P), as confirmed by quantitative RT-PCR (Fig 1C). Immunostaining analysis indicated reduced nuclear expression of *Cip1* and *Kip2* in OP9/L cells (Fig 1D). In agreement with the findings, OP9/L, but not OP9/P, cells showed significantly enhanced proliferation capacity compared to control OP9 cells (Fig 1E). It was also noted that such proliferating OP9/L cells showed an increased expression of CD34 (Fig 1E), which is associated with neovascularization in BM [22]. The results suggest that *Bcr-Abl* ^*+*^ leukemic cells specifically enhance the proliferation capacity of OP9 stroma cells by repressing *Cdkn* expression.

### KOBA cells enhance the proliferation capacity of OP9 cells by activating Notch signal

**Fig 2:**
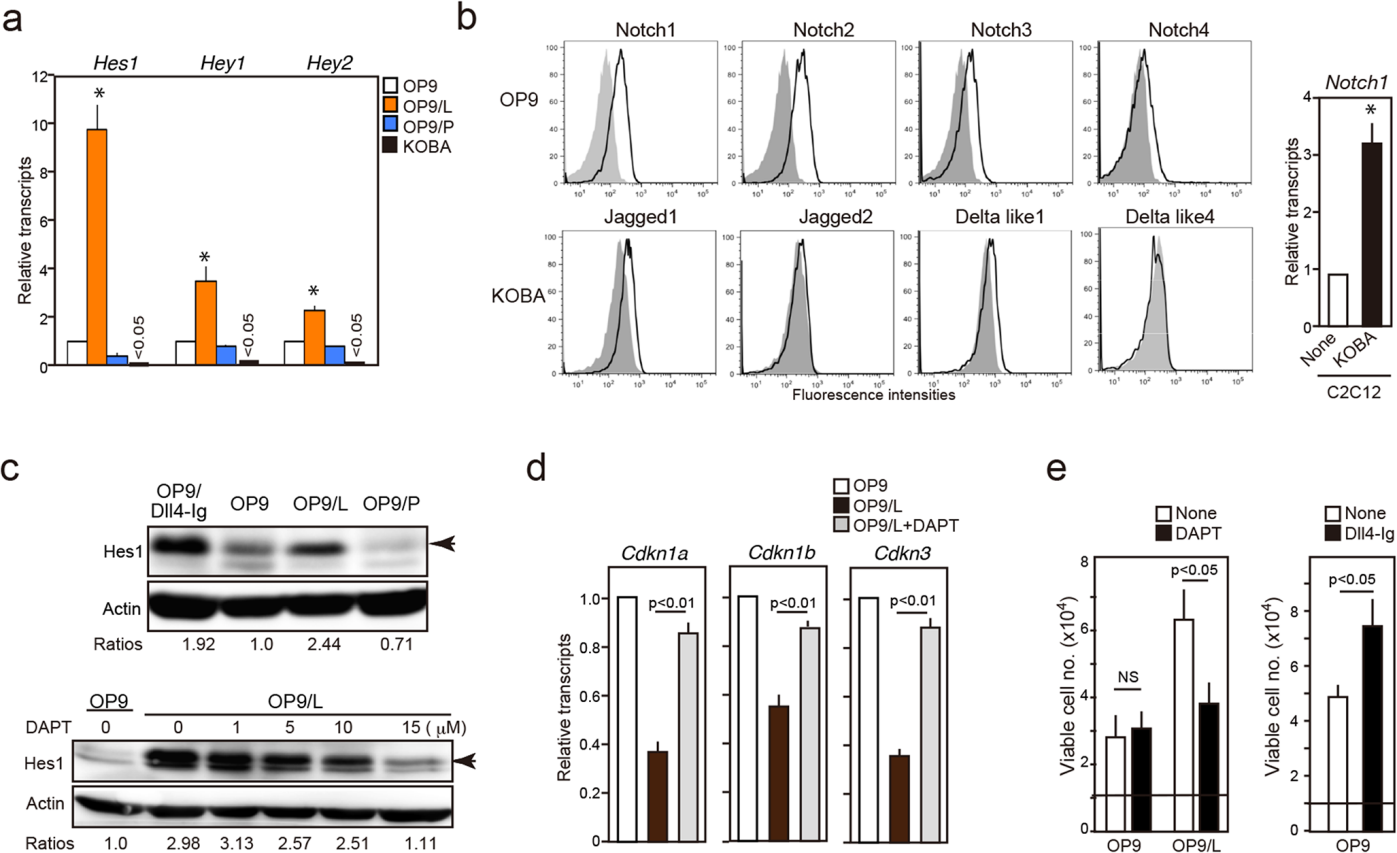
KOBA cells repress *Cdkn* expression and enhance proliferation of OP9 cells by Notch activation. (**a**) Expression of indicated Notch target genes was determined in OP9, OP9/L, OP9/P, and KOBA cells were determined using quantitative RT-PCR. The means and SEs of triplicate determination. *P < 0.01. (**b)** Cell surface expression of Notch receptors and ligands was analyzed in OP9 and KOBA cells, respectively, using FACS. Shaded areas indicate control staining (left). C2C12 myoblast cells were co-cultured with or without KOBA cells for 8 days, and *Notch1* transcripts in isolated C2C12 were determined by quantitative RT-PCR (right). The means and SEs of triplicate culture. *P < 0.05. (**c**) OP9, OP9/L, OP9/P, and OP9 cells cultured with coated Dll4-Ig protein were lysed and immunoblotted with anti-Hes1 and anti-actin antibodies (upper). Arrowhead indicate Hes1 signal at 31 kDa, and relative signal densities of Hes1 over actin are shown. OP9 cells were co-cultured with KOBA cells in the absence or continuous presence of varying concentrations of DAPT for 8 days, and the isolated OP9 cells were lysed and immunoblotted with anti-Hes1 antibody (lower). Arrowhead indicate Hes1 signal, and relative signal densities of Hes1 over Actin are shown. (**d**) Expression of indicated *Cdkn* genes was determined in OP9, OP9/L, and OP9/L induced in the presence of 15 μM DAPT. The means and SEs of triplicate determination. (**e**) OP9 cells (1 × 10^4^ cells) were cultured alone or with KOBA cells in the absence or presence of 15 μM DAPT for 8 days and the viable cell numbers were counted (left). OP9 cells (1 × 10^4^ cells) were also cultured with or without coated Dll4-Ig for 8 days, and the viable cell numbers were counted (right). The means and SEs of triplicate culture are shown.

OP9/L cells showed a remarkable increase in the Notch-target genes, *Hes1*, *Hey1*, and *Hey2*, compared with OP9 and OP9/P cells; the expression of these genes was negligible in KOBA cells and was not attributable to the minimal contamination by KOBA cells (Fig 2A). OP9 cells expressed Notch receptors, and KOBA cells expressed Notch ligands (Jagged1 and Dll1) (Fig 2B). Co-culture of KOBA cells with Notch-responsive C2C12 myoblast cells caused significant activation of *Notch1* in the C2C12 cells, indicating that the ligands on KOBA cells were functional (Fig 2B). We confirmed that OP9/L showed a higher expression of Hes-1 protein compared with OP9 and OP9/P cells, to the extent comparable with that in the OP9 cells stimulated with Dll4-Ig fusion protein (Fig 2C). Further, the *Hes-1* induction by the co-culture with KOBA cells was almost completely inhibited in the presence of a γ-secretase inhibitor (DAPT) at 15 μM (Fig 2C). We then examined the effects of DAPT on the *Cdkn* expression in OP9 cells. The repression of *Cdkn1a*, *Cdkn1b*, and *Cdkn3* by the co-culture with KOBA cells was abolished nearly completely in the presence of 15 μM DAPT (Fig 2D). Concordantly, enhancement of the proliferation capacity was also abrogated in the presence of DAPT, although the proliferation capacity of OP9 cells in the absence KOBA was unaffected (Fig 2E). We confirmed that the proliferation of OP9 cells was significantly enhanced in the presence of Dll4-Ig (Fig 2E). We also examined the reversibility of the effects. OP9 cells were co-cultured with KOBA cells for 13 days, and aliquots of the cultures were treated with 10 μM imatinib for 2 days from day 8 to 10, which killed essentially all KOBA cells without affecting OP9 cells in the culture. The increase of *Hes1/Hey1* and decrease of *Cdkn’s* were almost completely returned to the levels of control OP9 cells by the imatinib treatment (S3A Fig). The results strongly suggest that KOBA cells enhance the proliferation capacity of OP9 cells by directly activating Notch signal.

### KOBA cells induce altered expression of the ligands for integrins in OP9 cells

**Fig 3:**
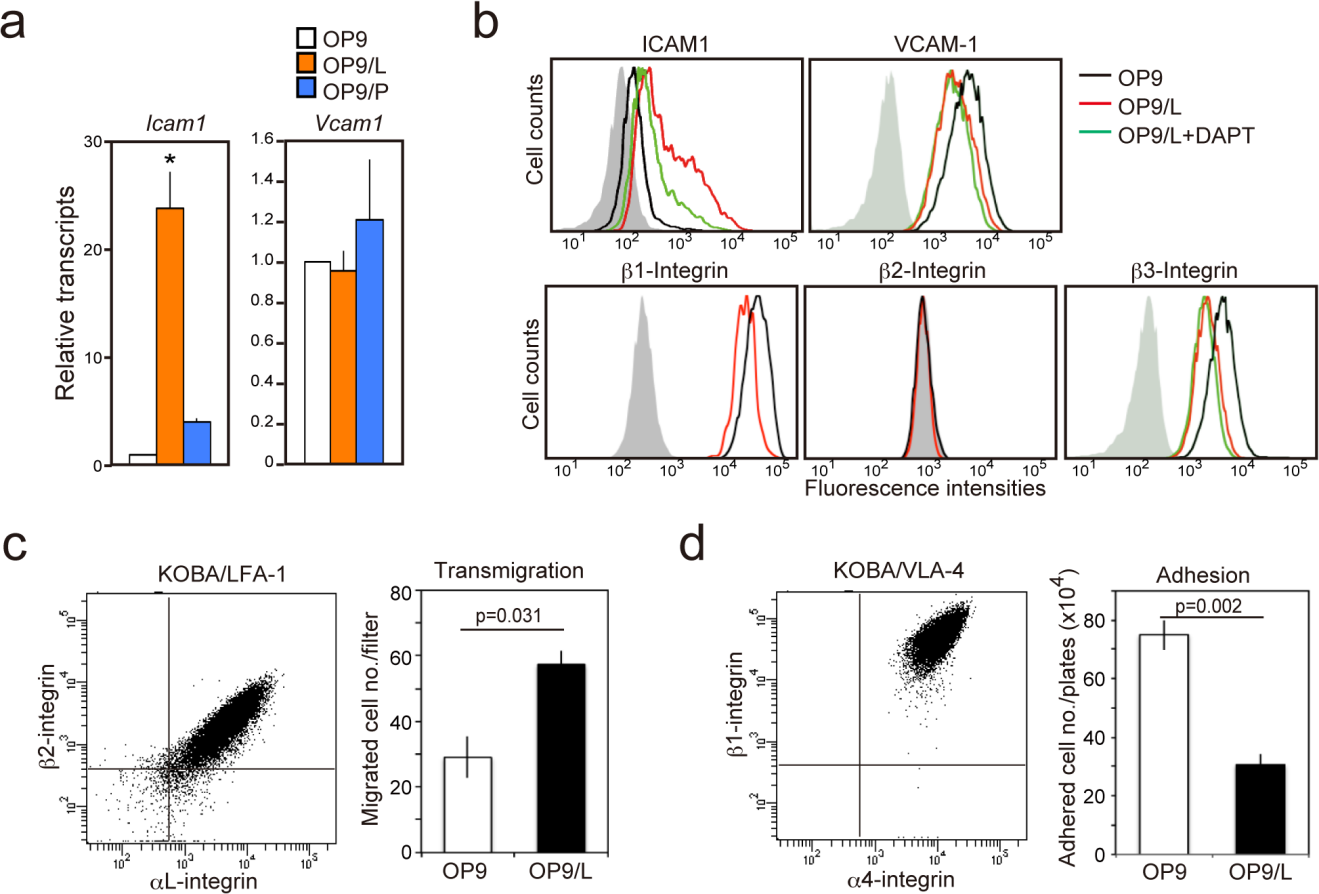
OP9/L cells show characteristic changes in integrin receptor expression and altered integrin-mediated interaction with KOBA cells. (**a**) The expression of *Icam1* and *Vcam1* in OP9, OP9/L, and OP9/L was determined by quantitative RT-PCR. The means and SEs of triplicate determination are shown. *P < 0.01. (**b**) OP9, OP9/L, and OP9/L (+DAPT) cells were analyzed for the surface expression of indicated molecules with FACS. Shaded areas indicate control staining. (**c**) Expression of LFA-1 on KOBA cells was analyzed with two-color FACS using anti-αL and anti-β2 integrin antibodies (left). KOBA cells were cultured on OP9 or OP9/L cell monolayers in a matrigel invasion chamber for 16 hours, and the cells that transmigrated the filters were counted (right). The means and SEs of triplicate culture are shown. (**d**) Expression of VLA-4 on KOBA cells was analyzed with two-color FACS using anti-α4 and anti-β1 integrin antibodies (left). KOBA cells were cultured on OP9 or OP9/L cell monolayers for 6 hours, and the cells that stably attached onto the monolayers were counted (right). The means and SEs of triplicate culture are shown.

OP9 cells express a high level of VCAM-1, a ligand for **α**4/β1 integrin, but only a minimal level of ICAM-1, a ligand for **α**L/β2 integrin. However, OP9/L, but not OP9/P, cells showed a remarkable increase in *Icam1* transcripts and accordingly, significantly increased cell-surface expression of ICAM-1 (Fig 3A and 3B). DAPT treatment significantly impaired the increase in ICAM-1 expression, suggesting the involvement of Notch activation (Fig 3B). In contrast, expression of VCAM-1 was rather decreased in OP9/L cells, and the effect was not affected by DAPT (Fig 3B). Since *Vcam1* transcripts were unaffected, the expression change of VCAM-1 was apparently posttranscriptional. The expressions of β1-integrin and β3-integrin were also reduced slightly (Fig 3B). We then examined the functional consequences of the altered expression of integrin ligands on the interaction with KOBA cells, which expressed both αL/β2 (LFA1) and α4/β1 (VLA-4) integrins (Fig 3C and 3D). KOBA cells showed significantly enhanced transmigration through OP9/L cells, probably mediated by LFA-1 [23], compared with OP9 cells (Fig 3C), whereas the stable adhesion to OP9/L cells, most likely via VLA-4 [24], was remarkably reduced (Fig 3D). The results indicate that the interaction of OP9 cells with KOBA cells renders the stroma cells more supportive for leukemia cell migration at the cost of static adhesion.

### KOBA cells induce a change in the cytokine gene expression pattern of OP9 cells

**Fig 4:**
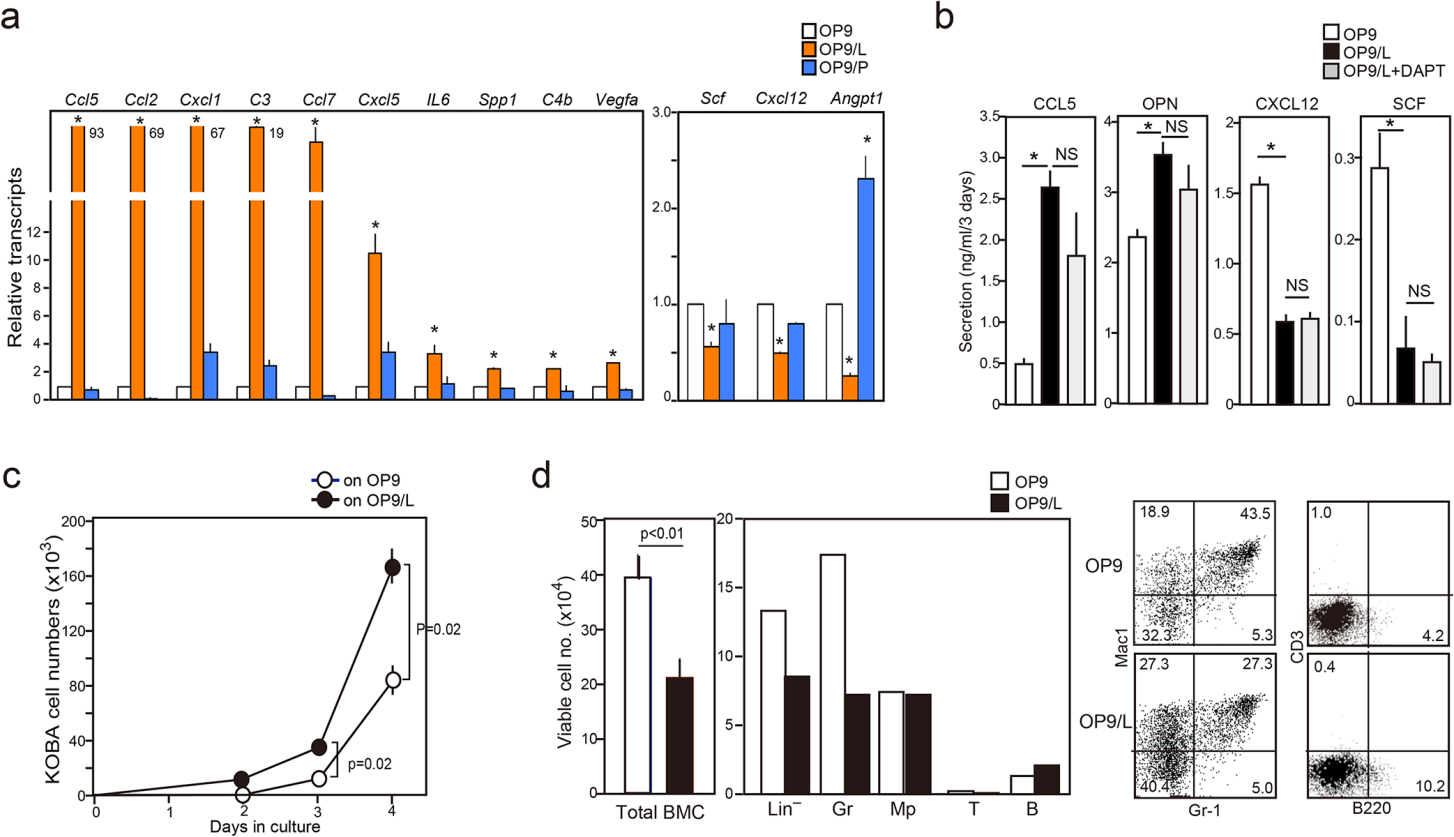
OP9/L cells show increased proinflammatory factor and decreased hematopoietic factor gene expression and enhance KOBA cell proliferation at the cost of supporting normal hematopoiesis. (**a**) OP9, OP9/L, and OP9/P cells were analyzed for the expression of indicated cytokine genes with quantitative RT-PCR. The means and SEs of triplicate determination are shown. *P < 0.01. (**b)** OP9, OP9/L, and OP9/L (+15 μM DAPT) cells were cultured for 3 days, and indicated cytokines in the culture supernatants were assessed with enzyme-linked immunosorbent assay. The means and SEs of triplicate culture are shown. *P < 0.05. (**c**) KOBA cells (1 × 10^3^ cells) were cultured with OP9 or OP9/L cell monolayers, and the viable cell numbers were counted on indicated days. The means and SEs of triplicate culture are shown. (**d**) Sorted lin^−^ BM cells (10^5^ cells) from normal B6 mice were cultured with OP9 or OP9/L cell monolayers for 5 days, and total recovered cell numbers were counted. An aliquot of the cells was analyzed with two-color FACS for indicated lineage markers, and the cell numbers of lin^−^ and mature hematopoietic cells were also assessed.

Notable changes in OP9/L cells were also detected in the expression of genes coding various cytokines. The OP9/L cells had a remarkable increase in the transcripts of proinflammatory factors, including various chemokines (*Ccl1*, *Ccl2*, *Ccl5*, *Ccl7*, *Cxcl1*, *Cxcl5*), complement elements, *IL-6*, *Spp1*, and *Vegfa*, compared with control OP9 cells (Fig 4A). In contrast, the expression of genes crucially involved in normal hematopoiesis, such as *Scf*, *Cxcl12*, and *Angpt1*, was significantly decreased (Fig 4A). Again, the effects were specific for KOBA cells, and OP9/P cells barely showed such changes, except for a rather increased *Angpt1* expression. We confirmed that OP9/L cells secreted significantly more Ccl5 and OPN and much less Cxcl12 and Scf than OP9 cells (Fig 4B). The altered cytokine pattern was unaffected by DAPT and was apparently independent of Notch activation (Fig 4B). Further, unlike the *Cdkn* repression via Notch activation, the effects on cytokine genes lasted longer, for at least 3 days after the depletion of KOBA cells with imatinib (S3B Fig). OP9/L cells showed significantly increased expression of a series of genes related to adipocyte differentiation including *Cebpb* and adipokines (S4 Fig) and thus, the diverse effects on cytokine expression may be partly associated with adipocytic differentiation of OP9 cells. We also investigated the functional consequences of the effects. The proliferation of KOBA cells was significantly enhanced in the presence of OP9/L cells compared to control OP9 cells (Fig 4C). In contrast, OP9/L cells showed significantly attenuated activity for supporting the hematopoiesis from normal lin^−^ BM cells, particularly the generation of mature granulocytes (Fig 4D). The results suggest that KOBA cells induce a markedly altered repertoire of cytokine production in OP9 cells, which contributes to the overall promotion of leukemia cell proliferation over normal hematopoiesis.

### KOBA cells differentially affect the proliferation and gene expression of primary ECs and MCs in vivo in BM

**Fig 5:**
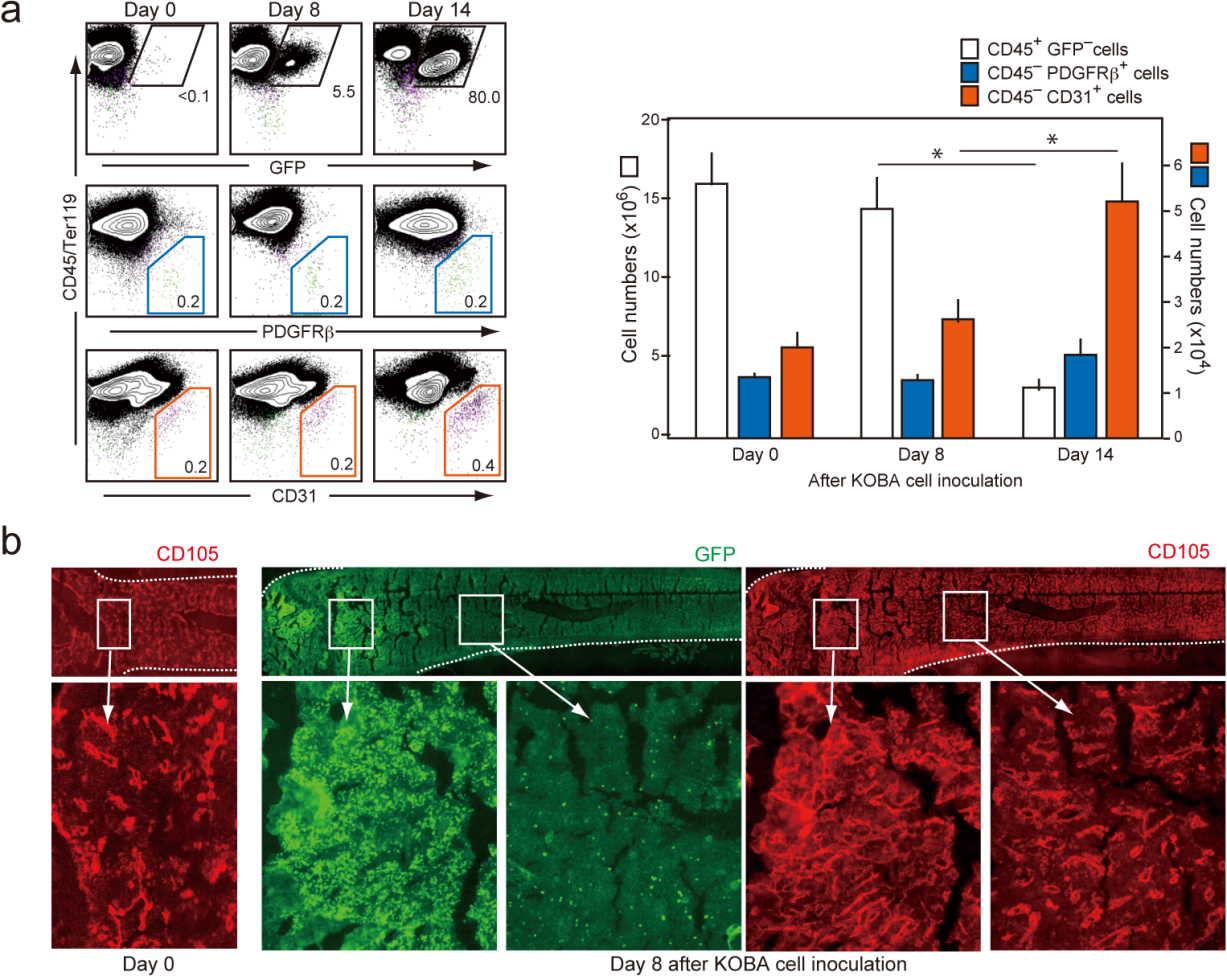
The numbers of ECs are increased around KOBA leukemia cells in BM. (**a**) Normal unirradiated B6 mice were injected intravenously with KOBA cells (3 × 10^3^ cells/mouse), and the BM cells were analyzed with two-color FACS for the expression of indicated molecules before and 8 and 14 days after the inoculation (left). The proportions of GFP^+^ KOBA cells, CD45^−^ PDGFRβ^+^ MCs, and CD45^−^ CD31^+^ ECs are indicated. The means and SEs of total GFP^−^ CD45^+^, EC, and MC cell numbers in five recipients were assessed (right). *P < 0.01. (**b**) The femoral bones of before and 8 days after KOBA inoculation were immunostained with anti-GFP and anti-CD105 antibodies. Enlarged images of the boxed regions are indicated.

**Fig 6:**
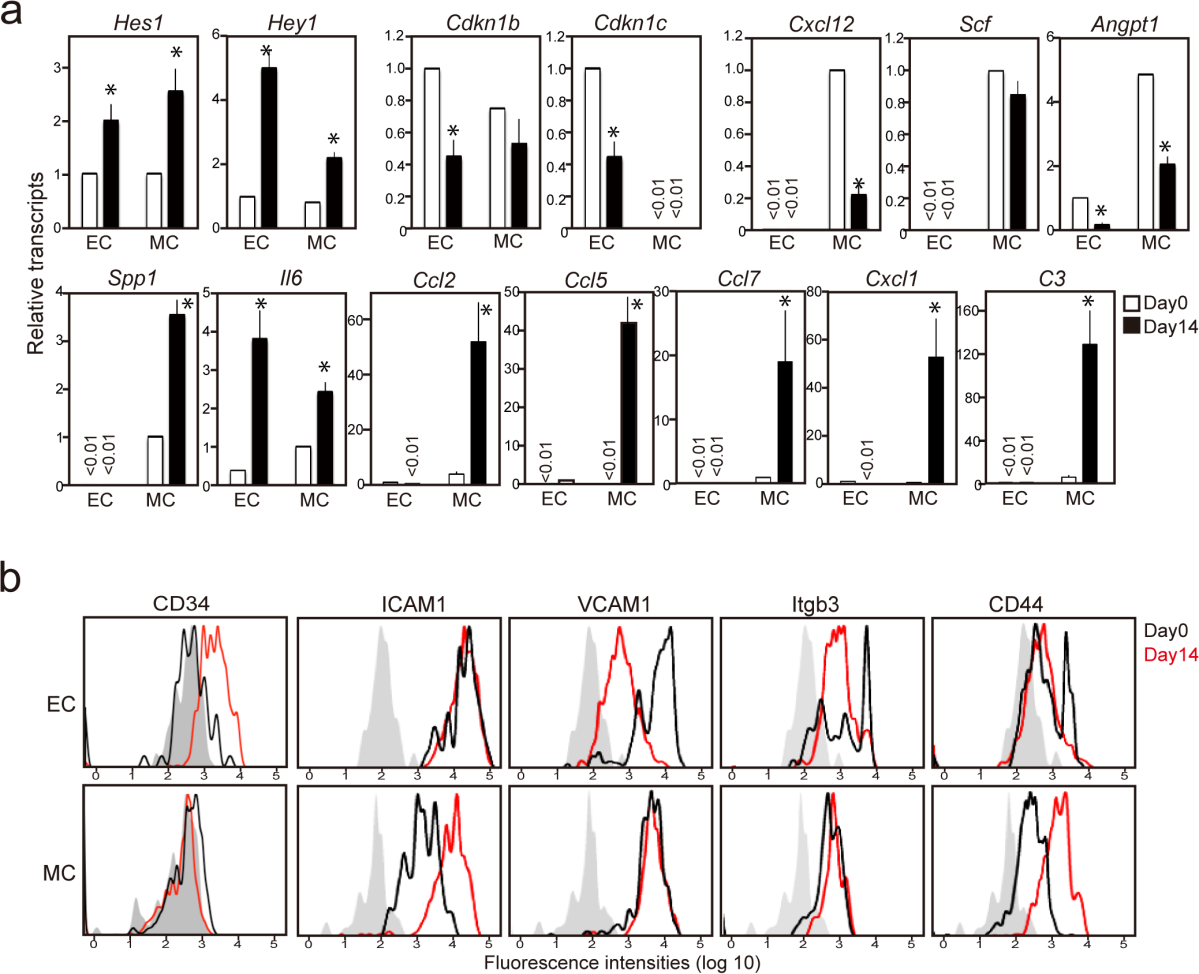
Leukemic invasion of KOBA cells induces distinctive effects on the gene expression and phenotypes of ECs and MCs in BM. (**a**) ECs and MCs were sorted from the BM of mice 14 days after KOBA cell inoculation, and the expression of indicated genes was determined with quantitative RT-PCR. The means and SEs from three mice are shown. *P* < 0.05. (**b**) The surface expression of indicated molecules was analyzed with multi-color FACS in the CD31^+^ (EC) and PDGFRβ^+^ (MC) cell gates before (black line) and 14 days after (red line) KOBA inoculation. Shaded areas indicate control staining.

We then examined the effects of KOBA cells on the primary stroma cells in BM by inoculating KOBA cells in normal, unirradiated mice (5 × 10^3^ cells per mouse). As GFP^+^ KOBA cells colonized and expanded in BM by day 14, the absolute numbers of GFP^−^CD45^+^ normal hematopoietic cells were profoundly decreased (Fig 5A). However, the total numbers of CD45^−^ PDFGRβ^+^ MCs were maintained, and those of CD45^−^ CD31^+^ ECs were even significantly increased (Fig 5A). Immunostaining analysis revealed that the ECs as detected with CD105 expression were increased locally in the regions coinciding with the leukemic cell foci at as early as 8 days (Fig 5B). Both ECs and MCs expressed Notch receptors and showed a significant increase in *Hes1* /*Hey1* expression compared with those in normal BM (S5 Fig and Fig 6A). Further, ECs, but not MCs, showed significantly decreased expression of *Cdkn1b* and *Cdkn11c* similar to OP9/L cells, which is consistent with the preferential increase in ECs (Fig 6A). On the other hand, MCs showed a reduced expression of *Cxcl12* and *Angpt1* and a remarkable increase in the expression of inflammatory cytokine genes, such as *Spp1*, *Il-6*, *Ccl2*, *Ccl7*, *Cxcl1*, and *C3*, again reminiscent of OP9/L cells (Fig 6A). ECs barely expressed these cytokine genes either in normal or in leukemic mice, except for *Il-6*, which was strikingly increased in the ECs of leukemic BM (Fig 6A). MCs and ECs also revealed distinct changes in the expression of cell-adhesion molecules. ECs showed increased CD34, which is characteristic of CML-associated angiogenesis in human, and decreased VCAM-1 and β3-integrin, whereas MCs exhibited a markedly increased expression of ICAM-1 and CD44 (Fig 6B). Altogether, these results suggest that the effects of KOBA cells on OP9 cells in vitro were differentially reflected on the primary ECs and MCs of BM in vivo during the leukemic invasion.

### Notch ligands on KOBA cells is involved in the increased ECs in BM and the leukemic expansion

**Fig 7:**
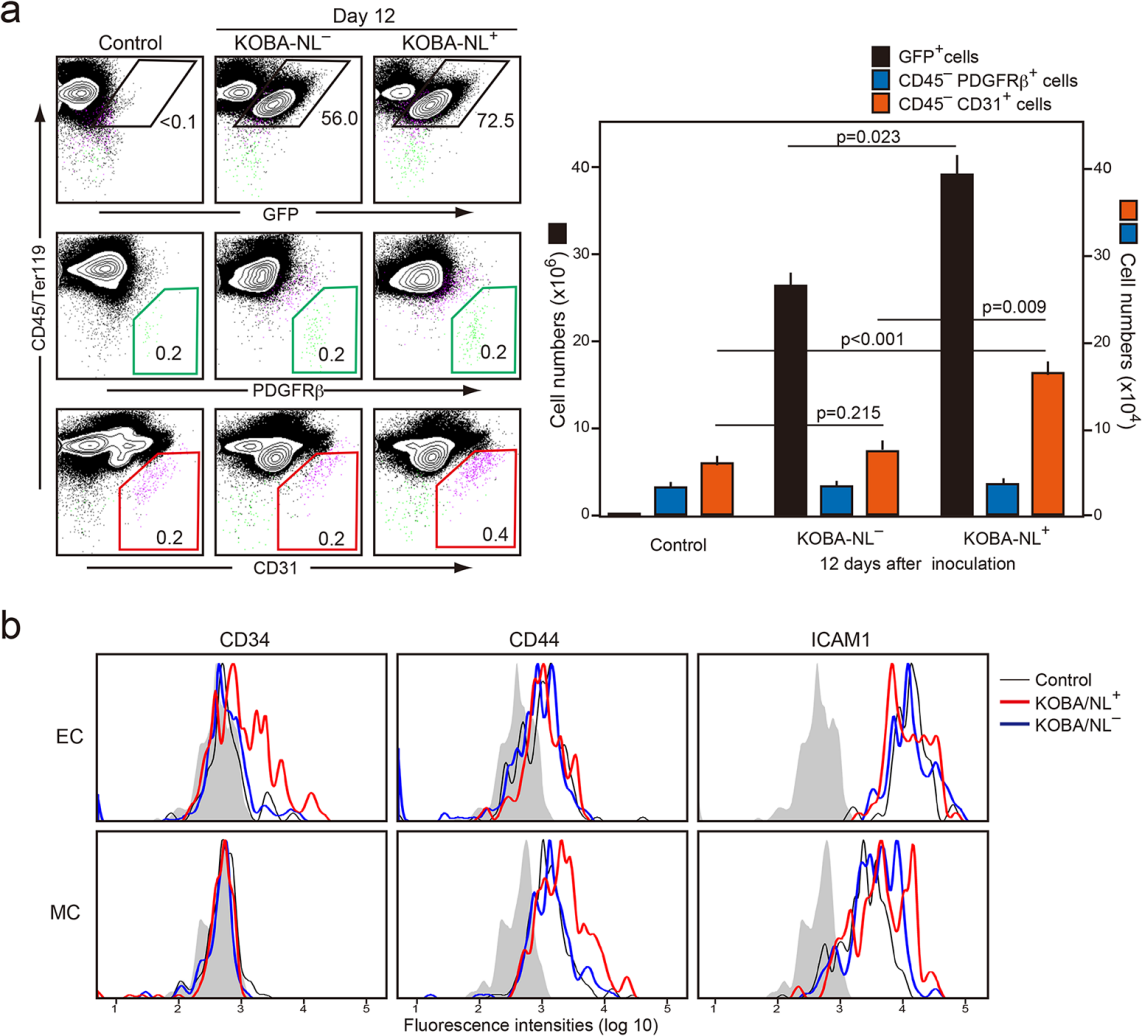
KOBA-NL^+^, but barely KOBA-NL^–^, cells induce the increase in EC numbers and altered expression of adherence molecules. **(a)** KOBA cells were separated into NL^+^ and NL^−^ fractions with a cell-sorter. Normal unirradiated B6 mice were injected intravenously with the sorted KOBA cells (3 × 10^3^ cells/mouse), and the BM cells were analyzed with two-color FACS for the expression of indicated molecules 12 days later (left). The proportions of GFP^+^ KOBA cells, CD45^−^ PDGFRβ^+^ MCs, and CD45^−^ CD31^+^ ECs are indicated. The means and SEs of total KOBA, EC, and MC cell numbers in 3 recipients were assessed (right). **(b)** The surface expression of indicated molecules was analyzed with multi-color FACS in the CD45^–^ CD31^+^ (EC) and CD45^–^ PDGFRβ^+^ (MC) cell gates 12 days after KOBA inoculation. Shaded areas indicate control staining.

To examine the involvement of Notch ligands (NLs) on KOBA cells in their leukemogenic activity, we separated the NL^−^ and NL^+^ KOBA cells with the use of a cell-sorter and inoculated them separately in normal mice (5x10^3^ cells per mouse). Co-culture of each fraction with OP9 cells in vitro confirmed that *Hes1/Hey1* activation and *Cdkn* repression in OP9 cells were observed only in the NL^+^ KOBA cells (S6 Fig). Although the sorted KOBA/NL^−^ cells gradually restored the NL expression during the propagation in culture, the levels remained to be lower than in KOBA/NL^+^ cells for at least 8 days at least in culture. Upon inoculation into normal mice, GFP^+^ KOBA/NL^+^ cells showed significantly more expansion in BM than KOBA/NL^−^ cells at day 12 (Fig 7A). The significant increase in ECs in BM was observed only in the KOBA/NL^+^-bearing mice (Fig 7A). The expression of CD34 in ECs as well as the increase in CD44 and ICAM1 expression in MCs were also detected in the mice inoculated with KOBA/NL^+^ cells (Fig 7B). These results suggest that the expression of NLs on KOBA cells contributes to the leukemogenic activity in vivo by affecting the microenvironmental stroma cells.

### Human CML cell lines alter the gene expression of OP9 cells similarly to KOBA cells

**Fig 8:**
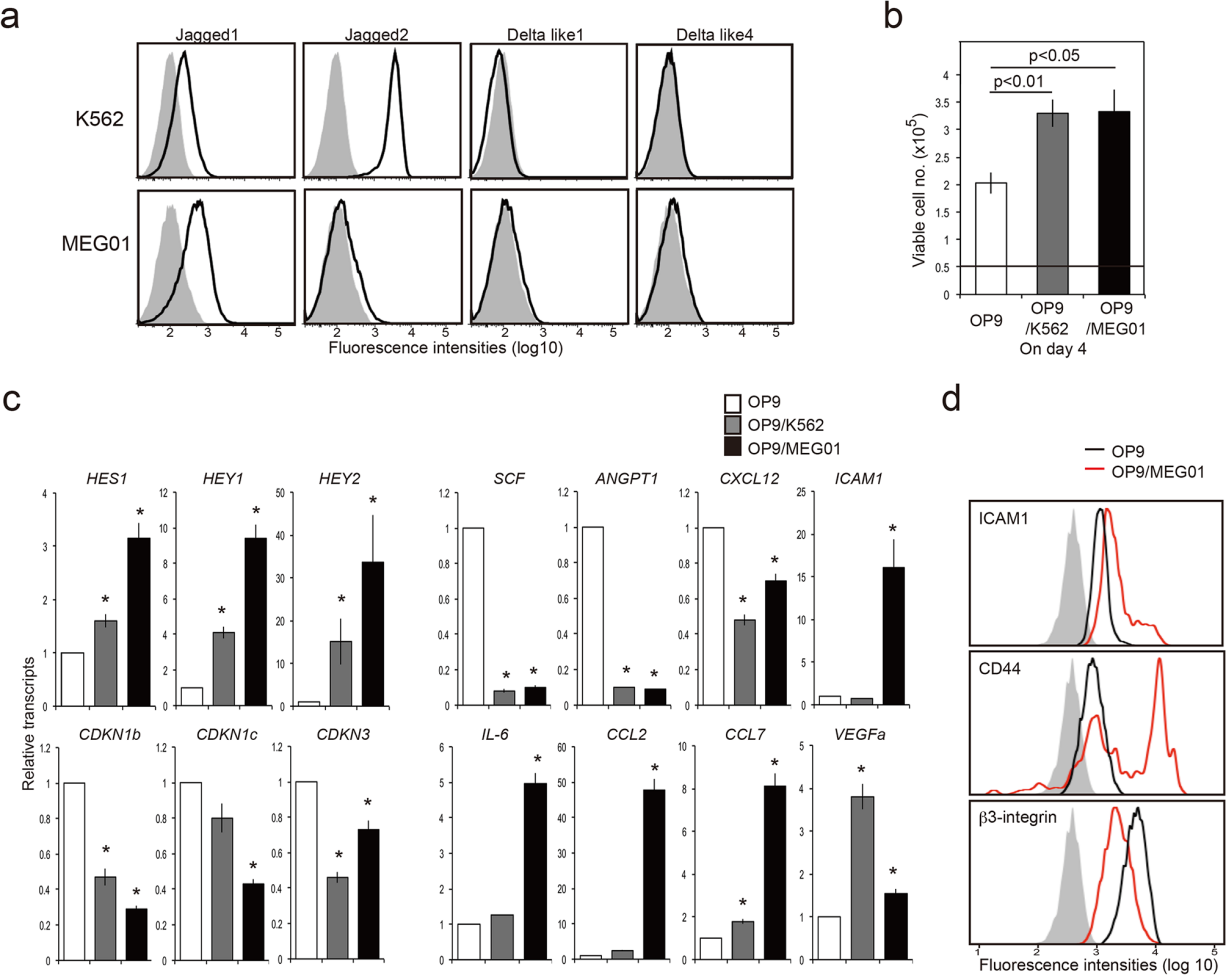
Human CML cell lines induce the changes in gene expression patterns of OP9 stroma cells on the co-culture. **(a)** K562 and MEG01, were analyzed for the expression of Notch ligands with FACS. Shaded areas indicate control staining. **(b)** OP9 cells were co-cultured with K562 or MEG01 cells. Eight days later, the CML cells were depleted, and the isolated OP9 cells were cultured (0.5×10^5^ cells) alone. The viable cell numbers were counted on day 4. **(c)** OP9 cells precultured alone, with K562, or MEG01 cells for 8 days were isolated, and the expression of indicated genes were analyzed with quantitative RT-PCR. The means and SEs of triplicate determination are shown. *P < 0.05. **(d)** OP9 cells precultured alone or with MEG01 cells for 8 days were isolated, and the expression of indicated molecules were analyzed with FACS.

We finally examined whether human CML cells also show the effects on stroma cells. It was confirmed that two independent CML cell lines, K562 and MEG01, expressed Notch ligands, Jagged 1 and Jagged 2 (Fig 8A). Taking the advantage of effective Notch interaction between human and murine cells [25], we co-cultured the human CML cells with OP9 cells. Both OP9/K562 and OP9/MEG01 cells showed significantly increased proliferation capacity compared to untreated OP9 cells (Fig 8B). Moreover, OP9/K562 and OP9/MEG01 cells showed markedly altered gene expression, including increased expression of Notch target genes (*Hes1*, *Hey1*, *Hey2*) and proinflammatory genes (*Il-6*, *Ccl2*, *Ccl7 and Vegfa*), and decreased expression of Cdk inhibitor genes (*Cdkn1b*, *Cdkn1c*, *Cdkn3*) and hematopoietic genes (*Scf*, *Angpt1*, *Cxcl12*) (Fig 8C). The effects were stronger in MEG01 cells than K562 cells. The OP9/MEG01 cells also exhibited increased ICAM1 and CD44 expression with reduced β3-integrin (Fig 8D). These results indicate that human CML cell lines can induce similar characteristic genetic changes in OP9 stroma cells.

## Discussion

In the current study, we investigated the effects of CML cells on the hematopoietic microenvironment. To examine the specific effects of *Bcr-Abl* on the interaction with stroma cells, we established a set of cell lines, a stroma-dependent, nonleukemic hematopoietic cell line (KOP1) and a KOP1 line transformed by *Bcr-Abl* expression (KOBA) and compared the effects on OP9 stroma cells representing MSCs.

The OP9 cells co-cultured with KOBA cells (OP9/L), but not with KOP1 cells (OP9/P), showed significant repression of *Cdkn* genes and increased proliferation capacity, and the effect was associated with the activation of Notch target genes including *Hes1*, *Hey1*, and *Hey2*. Further, OP9 and KOBA cells expressed Notchs and Notch ligands (NLs), most strongly Jagged1, respectively, and the *Cdkn* repression as well as the growth promotion was almost completely abrogated in the presence of a γ-secretase inhibitor (DAPT) during the co-culture. It is reported that Hes1 and Hey1 directly repress the transcription of *Cdkn* genes [26, 27], and thus, it is strongly suggested that the effect is mediated by Notch activation via KOBA cells. In addition, OP9/L cells showed a remarkable increase in *Icam1* transcripts with enhanced cell surface expression of ICAM-1, and the effect was also attenuated by DAPT. A recent report indicated that Notch1 stimulation of glioblastoma stem cells resulted in the enhanced expression of ICAM-1 [28]. In agreement with the findings, KOBA cells expressing **α**L/β2-integrin, a receptor for ICAM-1, exhibited much more efficient transmigration through OP9/L cells than through OP9 cells. These results suggest that the *Bcr-Abl* ^*+*^ leukemia cells actively modify the proliferation capacity and function of OP9 stroma cells by activating Notch signal.

OP9/L cells also showed characteristic changes in the expression of a wide range of cytokine genes. Thus, OP9/L cells expressed markedly reduced transcripts of *Scf*, *Cxcl12*, and *Angpt1* and secreted less amounts of SCF and CXCL12, which are crucial for supporting normal hematopoiesis [6, 29], than parental OP9 cells. In concordance with the findings, OP9/L cells showed significantly attenuated activity to support the proliferation and differentiation of normal lin^−^ BM cells, particularly granulocyte development, in vitro. The decreased expression of VCAM-1, β1-integrin, and β3-integrin in OP9/L cells may additionally contribute to the effects [30, 31]. In contrast, OP9/L cells revealed a remarkably increased expression of many proinflammatory cytokine genes, including various chemokine genes, *IL6*, *Spp1*, and *Vegfa*, compared to the parental OP9 and OP9/P cells. Accumulating evidence indicates that a proinflammatory microenvironment favors the expansion of leukemic cells including CML [16, 32], and an important role of IL-6 in the progression of CML has also been reported [16, 33]. We found that OP9/L cells supported the KOBA cell proliferation significantly better than control OP9 cells in vitro, and our preliminary results indicate that the culture supernatant of OP9/L cells alone was capable of enhancing their proliferation (Supper E and Minato N, unpublished observations). The change in the cytokine gene expression pattern was resistant to DAPT treatment and was apparently Notch-independent. It is reported that CXCL12-abundant reticular (CAR) cells, which are crucial for normal hematopoiesis, represent mesenchymal progenitors with the potential for terminal differentiation into adipocytes and chondrocytes [34]. Because OP9/L cells showed increased expression of *Cebpb* and several adipokine genes, it may be possible that the altered cytokine pattern is related to the differentiation induction via KOBA cells. The assumption may be consistent with the finding that the changes in cytokine genes lasted longer after KOBA cell removal than Notch-mediated effects. In any case, these results suggest that KOBA cells induce a functional remodeling of OP9 stroma cells, which benefits the leukemia cell proliferation at the cost of normal hematopoiesis.

Such characteristic changes in the gene expression of OP9/L cells were recapitulated in primary stroma cells after KOBA cell invasion in BM, although the effects were quite distinctive in ECs and MCs. The number of ECs was markedly increased around the leukemic cell foci in BM. The ECs showed a significant increase in *Hes/Hey1* expression along with the repression of *Cdkn1b*/*Cdkn1c*, which is consistent with Notch activation. Indeed, the effects were hardly observed when sorted KOBA/NL^−^ cells were inoculated, indicating that the hypervascularity was attributed to Notch activation in ECs via leukemia cells. Similar to OP9/L cells, the primary ECs in KOBA-bearing mice showed increased CD34 expression, which is characteristic of new angiogenesis in human CML [35], implying that these ECs are derived in part from MSCs via transdifferentiation [36] [37]. The effects of KOBA invasion on the expression of cell-adhesion molecules were also distinct between ECs and MCs in vivo. Among the changes observed in OP9/L cells, the increase in ICAM1 and CD44 expression was detected exclusively in MCs, whereas the reduced expression of VCAM-1 and β3-integrin was seen only in ECs. Although both ECs and MCs showed the activation of *Hes1/Hey1*, it appears that the consequences of Notch activation vary in the context of stroma cell types. Importantly, KOBA/NL^+^ cells expanded more efficiently than KOBA/NL^−^ cells in BM, suggesting that the Notch-mediated modification of BM stroma cells plays a significant role in the leukemic cell expansion (S7 Fig).

The prominent changes in the cytokine gene expression observed in OP9/L cells, including decreased hematopoietic genes and a remarkable increase in proinflammatory genes, were observed almost exclusively in MCs. The profound decrease of Cxcl12 is consistent with the deregulation of normal hematopoiesis commonly associated with CML [38]. Ccl2 and Ccl5 have been reported to be involved in the mobilization and homing of MCs to tumor sites and to participate in the maintenance of a proinflammatory state that favors tumor growth as well as metastatic colonization [39, 40]. An only exception was *Il-6*, which is reported to be activated by CML cells and to play an important role in CML development [16], and ECs showed a remarkable increase in *Il-6*, even more so than MCs. Such an altered cytokine milieu in BM may favor CML promotion over normal hematopoiesis (S7 Fig).

We demonstrated that human CML cell lines also expressed NLs and induced the changes in the gene expression of OP9 stroma cells essentially identical to those by murine KOBA cells. Thus, MEG01, and to less extent K562, cells induced a potent activation of Notch-target genes and caused repression of *Cdkns* and activation of *Icam1*in OP9 cells by the co-culture. The CML cells also induced a marked repression of hematopoietic genes with a concomitant increase of proinflammatory cytokine genes. It would be quite feasible that CML cells induce the remodeling of BM microenvironment favoring the CML cell proliferation and spread in humans as well. Intervening the effects of CML cells on BM stroma cells may provide an alternative therapeutic target controlling human CML.

## Materials and Methods

### Mice

C57BL/6 (B6) mice at the ages of 8 to 10 weeks were purchased from Japan SLC, Kyoto, Japan. *Sipa1* ^*−/−*^ mice [20] and B6 mice were maintained in specific pathogen-free conditions at Laboratory Animals Center, Kyoto University in accordance with the Institutional Animal Care and Use Committee (IACUC) Guidelines of Kyoto University Graduate School of Medicine. The mice were sacrificed by spinal cord dislocation under anesthesia with isoflurane. The protocol was approved by the IACUC of Kyoto University Graduate School of Medicine. All efforts were made to minimize suffering.

### Cell lines

The KOP1 cell line was established from the BM of 8-month-old *Sipa1* ^*−/−*^ mice with no myeloproliferative disease. The BM cells were cultured in complete BXH2 medium (Dulbecco’s modified Eagle’s medium [DMEM] supplemented with 10% heat-inactivated fetal calf serum, 10% NTC-109, 50 μM 2-mercaptoethanol, 100 μM nonessential amino acids, 1 mM sodium pyruvate, and antibiotics) for 2 weeks, and the cobblestone-like cells were passaged on OP9 cell monolayers every week to develop a continuous cell line. The KOP1 cell line was infected with a *pMCs-ires-EGFP* retroviral vector containing *p210 Bcr-Abl* cDNA as previously reported [19] (ref. 19), to establish the KOBA cell line. OP9 stroma cells were maintained in α-MEM (Invitrogen, Carlsbad, CA) supplemented with 20% etal bovine serum (FBS). The Notch-responsive C2C12 cell line was maintained in DMEM supplemented with 10% FBS. The human CML cells lines, K562 and MEG01, were kindly provided by Dr. A. Takaori, Department of Hematology, Graduate School of Medicine, Kyoto University, Japan, and were maintained in RMPI supplemented with 10% FBS.

### Cell cultures

KOP1 or KOBA cells (10^6^ cells) were co-cultured with OP9 cell monolayers in 10-cm dishes in complete BXH2 medium. After 5 days, overgrown KOBA cells were depleted with gentle pipetting from the culture every 2 days. After 8 days, the cultures were treated with trypsin/EDTA and stained with FITC-anti-CD45 antibody, and then CD45^−^ OP9 cells were isolated using FACSAria II (BD Biosciences) or AutoMax (Miltenyl Biotec, Bergisch Gladbach, Germany). In the OP9 cells co-cultured with KOP1 and KOBA cells were called OP9/P and OP9/L, respectively. For in vitro hematopoiesis, B6 BM cells were stained with a mixture of lineage markers (anti-CD45, anti-Ter119 [Biolegend] anti-CD3, anti-CD4, anti-CD8, anti-Mac-1, anti-Gr-1, anti-B220, and anti-NK.1.1 [BD Biosciences]), and the lin^−^ cells were isolated using AutoMax and cultured on OP9 or OP9/L cell monolayers for 5 days. For the Notch ligand assay, C2C12 cell monolayers were cultured in the absence or presence of KOBA cells for 2 days, and the CD45^−^ C2C12 cells were isolated using AutoMax. K562 or MEG-01 cells (10^6^ cells) were co-cultured with OP9 cell monolayers in 10-cm dishes in RPMI medium. After 8 days, the cultures were treated with trypsin/EDTA, stained with human FITC-anti-CD45RA and APC-anti-CD45RO antibody, and CD45^−^ OP9 cells were isolated using AutoMax (Miltenyl Biotec, Bergisch Gladbach, Germany).

### Flow cytometry

Flow cytometric analysis of the OP9 cells and primary BM cells were performed with the use of FACSCanto (Becton Dickinson, San Jose, CA). The following antibodies were used: anti-PDGFRβ, anti-CD31, anti-CD45, anti-Ter119, anti-CD54 (Biolegend), anti-CD3, anti-CD4, anti-CD8, anti-CD34, anti-Mac-1, anti-Gr-1, anti-B220, and anti-TER-119, anti-NK.1.1, anti-CD106 anti-hCDRA (BD Biosciences), and anti-CD61 anti-CD140a (eBioscience) anti-CD44 anti-hCD45RO (Pharmingen), anti-Notch-1, anti-Notch-4, anti-Jagged-1 (eBioscience), anti-Notch-2, anti-Notch-3, anti-Dll-1, and anti-jagged-2 (Biolegend).

### DNA microarray analysis

RNA was extracted from OP9 and purified OP9/L cells with Trizol and subjected to DNA microarray analysis in oligomer chips covering 20,000 genes by TaKaRa Bio Inc. (Siga, Japan). Data were filtered to remove low-confidence measurements and were globally normalized per array.

### Quantitative PCR

Total RNA was extracted with Trizol extraction followed by cDNA synthesis with SuperScript III (Invitrogen). Real-time PCR was performed with a LightCycler SYBR Green I marker kit on a LightCycler instrument (Roche, Basel, Switzerland). Cyclosporin served as an internal control. The primer pairs are listed in S2 Table.

### Immunoblotting and immunostaining

Cells were lysed with RIPA buffer (150 mM NaCl, 25 mM Tris-HCl [pH 7.6], 1% NP-40, 1% sodium deoxycholate, 0.1% sodium dodecyl sulfate, protease, and phosphatase inhibitors) and immunoblotted with anti-p27/Kip1 (Cell Signaling Technology, Beverly, MA, USA), anti-Hes-1, and anti-actin (Santa Cruz Biotechnology, Santa Cruz, CA, USA). For immunostaining of OP9 cells, the cells cultured on cover slips were fixed with 3% formalin, permeabilized with 0.02% Triton, blocked with 4% bovine serum albumin (BSA) in phosphate-buffered saline (PBS), and incubated overnight with anti-p21/Cip1 or anti-p57/Kip2 antibody (Upstate Co., Billercia, MA, USA), followed by Cy3-conjugated anti-mouse or Cy3-conjugated anti-rat (Jackson ImmunoResearch) IgG, along with DAPI. The images were visualized using a ZEISS Axiovert200M microscope equipped with an Axiocam MRm camera (Carl Zeiss MicroImaging, Göttingen, Germany). For staining BM, the femoral bones were fixed in 2% paraformaldehyde and left overnight in 30% sucrose before being snap-frozen in optimum cutting temperature compound; frozen sections were fixed with acetone. After blocking with BSA (1% in PBS), samples were stained with anti-GFP and anti-CD105 (eBioscence) antibodies followed by secondary antibodies Cy3-conjugated anti-rat IgG and Alexa Fluor 488–conjugated anti-rabbit IgG (Invitrogen) along with DAPI, and mounted in Mowiol (Calbiochem). Images were acquired with the use of fluorescence microscopy (Axiovert 200M) equipped with an AxioCam MRm Fluar 2.5×/0.12 or 5×/0.25 numerical aperture objective lens (Carl Zeiss) and analyzed with AxioVision version 4.6 (Carl Zeiss). Digital images were processed using Adobe Photoshop CS2 (Adobe Systems).

### Cell migration and adhesion assay

The migration potential of KOBA cells was evaluated using a BD BioCoat Matrigel Invasion Chamber. KOBA cells (10^5^ cells) were plated on OP9 or OP9/L monolayers in a matrigel invasion chamber and incubated for 16 hours. The under-surfaces of the filters were gently rinsed with PBS followed by fixation in 100% methanol and stained with Giemsa solution. The samples were examined with a microscope (Carl Zeiss) and the numbers of KOBA from the OP9/L co-culture cells per field in three wells were counted. For cell adhesion, KOBA cells (10^6^ cells) were cultured for 6 hours on OP9 or OP9/L cell monolayers at 37°C in BXH2 medium. The floating cells were aspirated and gently rinsed with warm PBS twice to eliminate the weakly attached KOBA cells. The cultures were treated with trypsin/EDTA, and the remaining KOBA cells were counted.

### Reagents

The γ-secretase inhibitor (GSI) (DAPT, Calbiochem, San Diego, CA) and Imatinib mesylate (Gleevec, STI571, Novartis) were obtained commercially.

### Statistical analysis

Statistical analysis was performed using a Student *t*-test.

## Supporting Information

S1 Fig KOP1 cell line represents immature hematopoietic cells, whose survival is absolutely dependent on OP9 stroma cells. (a) KOP1 cells cultured in the presence of OP9 cells were immunostained with indicated antibodies (left), and those separated from OP9 cells were cytospun and stained with Giemza solution (right). (**b**) KOP1 cells were two-color analyzed for the expression of indicated markers with FACS. (**c**) Genomic PCR analysis of *Sipa1* and *neo* in the gene targeting vector of KOP1 as well as B6 and *Sipa-1* ^*–/–*^ BM cells. (**d**) KOP1 cells were detached from OP9 cells, cultured in the absence of OP9 stroma cells for indicated periods, and were analyzed for the expression of indicated markers (upper) as well as DNA contents (lower). The progressive increase in apoptotic cells is noted with little change in the cell cycle pattern of viable cells.
(TIF)

S2 Fig Expression of *Bcr-Abl* in KOP1 cells results in the potent leukemogenic activity in vivo. Normal B6 mice (14 mice/group) were inoculated with KOP1 (10^7^ cells/mouse) or KOBA (10^4^ cells/mouse) cells, and the survival rates were examined.
(TIF)

S3 Fig The effects of KOBA cells on the cytokine gene expression of OP9 cells last longer than the Notch-mediated effects. OP9 cells were cultured in the absence of presence of KOBA cells for 13 days, with depletion of overgrown KOBA cells every 2 days. At day 8, imatinib (10μM) was added in aliquots of cultures for 2 days, and then replaced with fresh medium. On day 13, the OP9 cells were recovered by depleting KOBA cells, and the transcripts of indicated genes were examined with qRT-PCR. The vast majority of KOBA cells died in 24 hours after imatinib addition, whereas OP9 cells were barely affected. The means and SE of triplicate determination are indicated.
(TIF)

S4 Fig OP9/L cells show increased expression of adipocyte-related genes. Expression of indicated genes was determined in OP9, OP9/L, and OP9/P cells using quantitative RT-PCR. The means and SEs of triplicate determination are shown. *P < 0.05.
(TIF)

S5 Fig Both primary ECs and MCs in BM express Notch receptors. Cell surface expression of Notch receptors was analyzed for primary ECs and MCs from BM with FACS. Shaded areas indicate control staining.
(TIF)

S6 Fig NL^+^, but barely NL^–^, KOBA cells are capable of inducing *Hes1/Hey1* and repressing *Cdkn’s* of OP9 stroma cells. KOBA cells were stained with the mixture of anti-NL (Jagged1, Jagged2, Dll-1) antibodies, and the NL^+^ and NL^−^ fractions were sorted as indicated with FACS AriaIII. Each fraction was cultured in the presence of OP9 cells for 8 days, OP9 cells were recovered after depleting CD45^+^ KOBA cells with AutoMax, and the expression of indicated genes were determined by quantitative RT-PCR. The means and SEs of triplicate determination are shown.
(TIF)

S7 Fig Bcr-Abl^+^ leukemia cells differentially affect the gene expression of ECs and MCs in BM. Bcr-Abl^+^ leukemia cells directly activate Notch signal, leading to the repression of Cdk inhibitor genes in ECs and neovasculogenesis. It is possible that the increase in ECs also involves the transdifferentiation from MSCs associated with CD34 expression. Notch activation in MCs causes increased ICAM1 expression, promoting the leukemia cell migration. Also, Bcr-Abl^+^ leukemia cells repress the hematopoietic genes but remarkably enhance the expression of diverse proinflammatory genes in MCs. The effects are Notch-independent and may involve the differentiation promotion to adipocytes. Such a drastic change in the cytokine milieu may favor the expansion of leukemia cells at the cost of normal hematopoiesis in the BM. HSPC; hematopoietic stem/progenitor cell.
(TIF)

S1 Table Altered gene expression in OP9 cells by the coculture with KOBA. (PDF)

S2 Table List of the primer pairs (PDF)
