# Supplementary figures and images for: Modification of Gene Expression, Proliferation, and Function of OP9 Stroma Cells by Bcr-Abl-Expressing Leukemia Cells
Source: PLoS One. 2015 Jul 28;10(7):e0134026. doi: 10.1371/journal.pone.0134026 (PMC4517910; doi:10.1371/journal.pone.0134026)

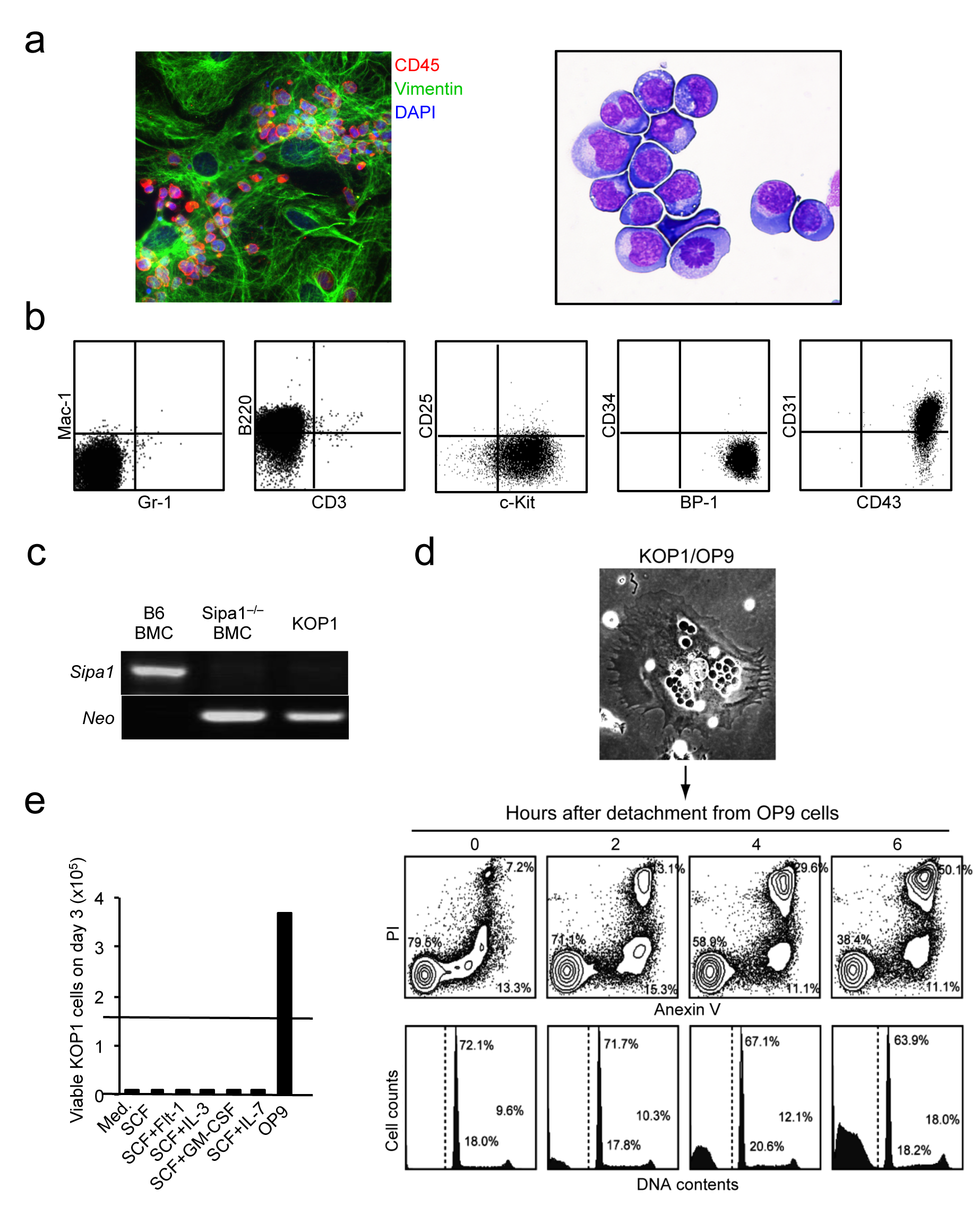

Supplement: S1 Fig — (a) KOP1 cells cultured in the presence of OP9 cells were immunostained with indicated antibodies (left), and those separated from OP9 cells were cytospun and stained with Giemza solution (right). (b) KOP1 cells were two-color analyzed for the expression of indicated markers with FACS. (c) Genomic PCR analysis of Sipa1 and neo in the gene targeting vector of KOP1 as well as B6 and Sipa-1 –/– BM cells. (d) KOP1 cells were detached from OP9 cells, cultured in the absence of OP9 stroma cells for indicated periods, and were analyzed for the expression of indicated markers (upper) as well as DNA contents (lower). The progressive increase in apoptotic cells is noted with little change in the cell cycle pattern of viable cells. (TIF) [file pone.0134026.s001.tif]

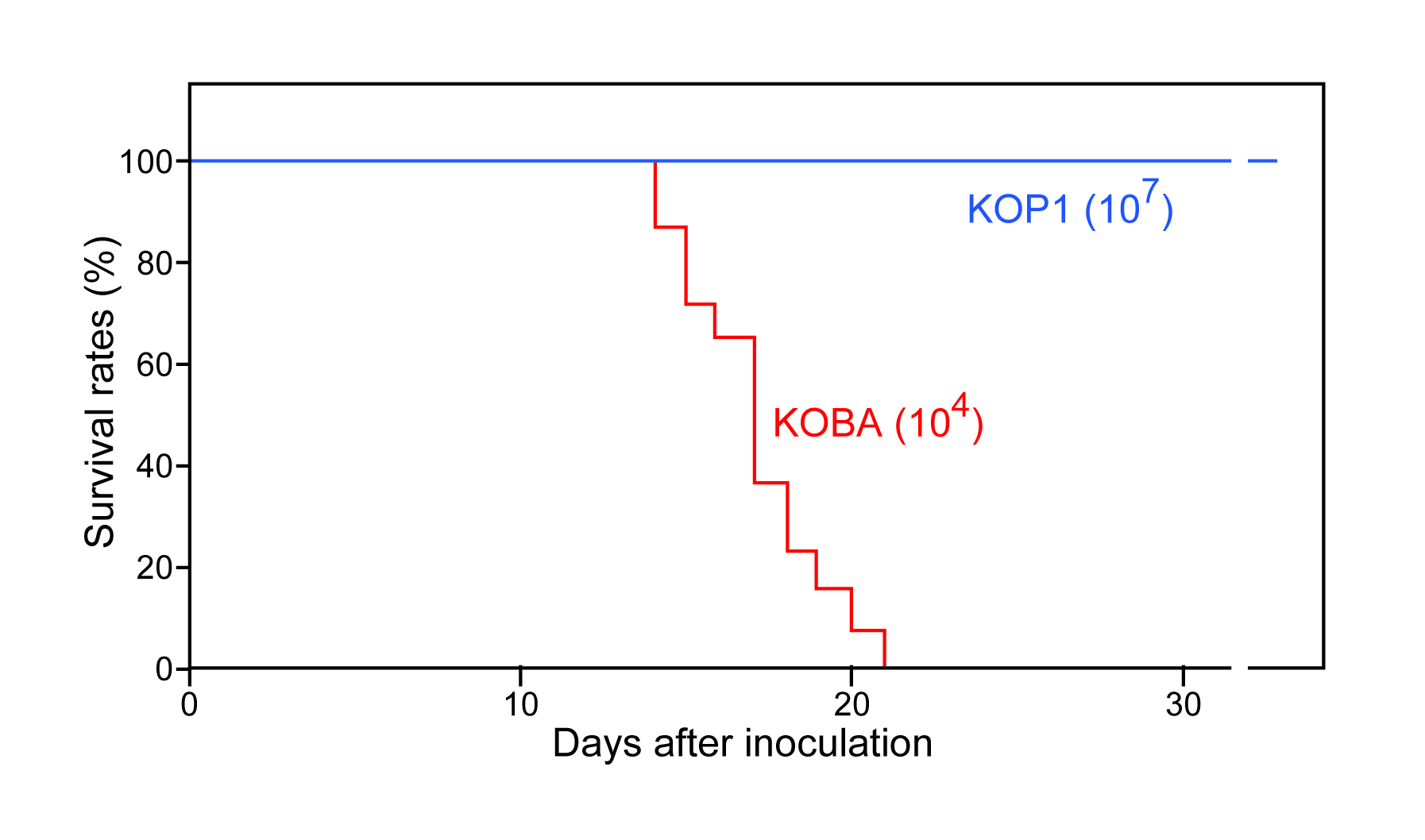

Supplement: S2 Fig — Normal B6 mice (14 mice/group) were inoculated with KOP1 (107 cells/mouse) or KOBA (104 cells/mouse) cells, and the survival rates were examined. (TIF) [file pone.0134026.s002.tif]

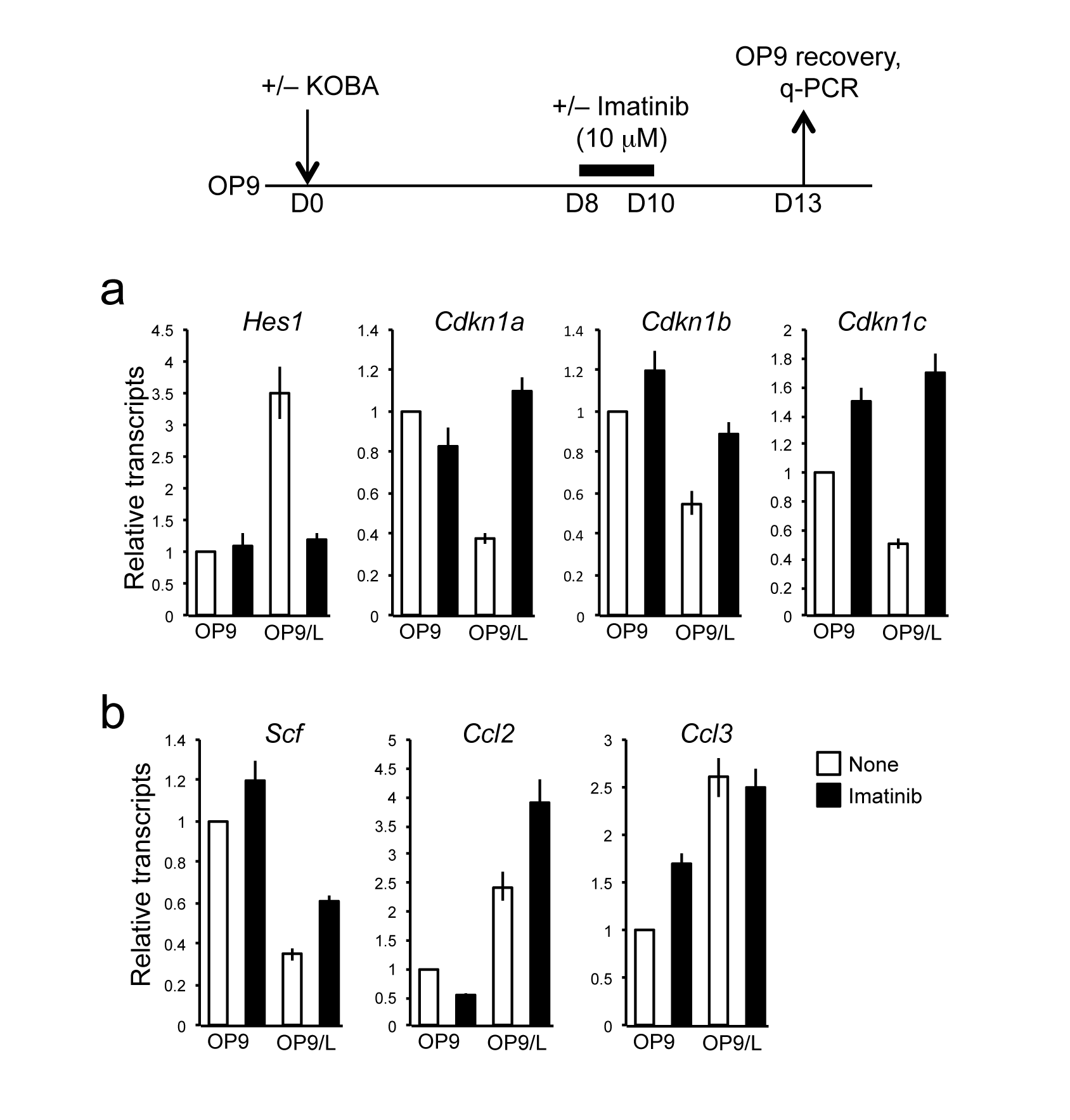

Supplement: S3 Fig — OP9 cells were cultured in the absence of presence of KOBA cells for 13 days, with depletion of overgrown KOBA cells every 2 days. At day 8, imatinib (10μM) was added in aliquots of cultures for 2 days, and then replaced with fresh medium. On day 13, the OP9 cells were recovered by depleting KOBA cells, and the transcripts of indicated genes were examined with qRT-PCR. The vast majority of KOBA cells died in 24 hours after imatinib addition, whereas OP9 cells were barely affected. The means and SE of triplicate determination are indicated. (TIF) [file pone.0134026.s003.tif]

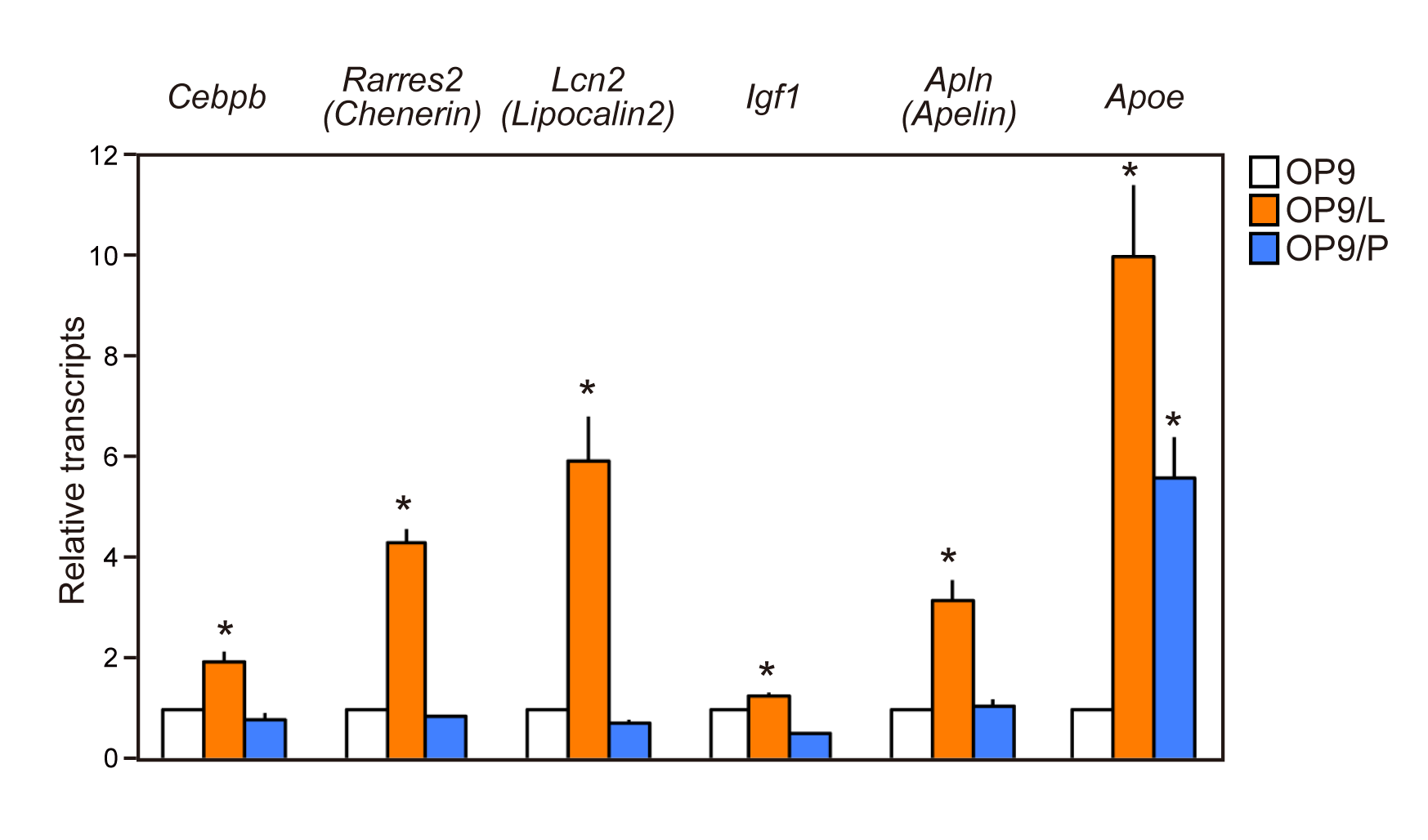

Supplement: S4 Fig — Expression of indicated genes was determined in OP9, OP9/L, and OP9/P cells using quantitative RT-PCR. The means and SEs of triplicate determination are shown. *P < 0.05. (TIF) [file pone.0134026.s004.tif]

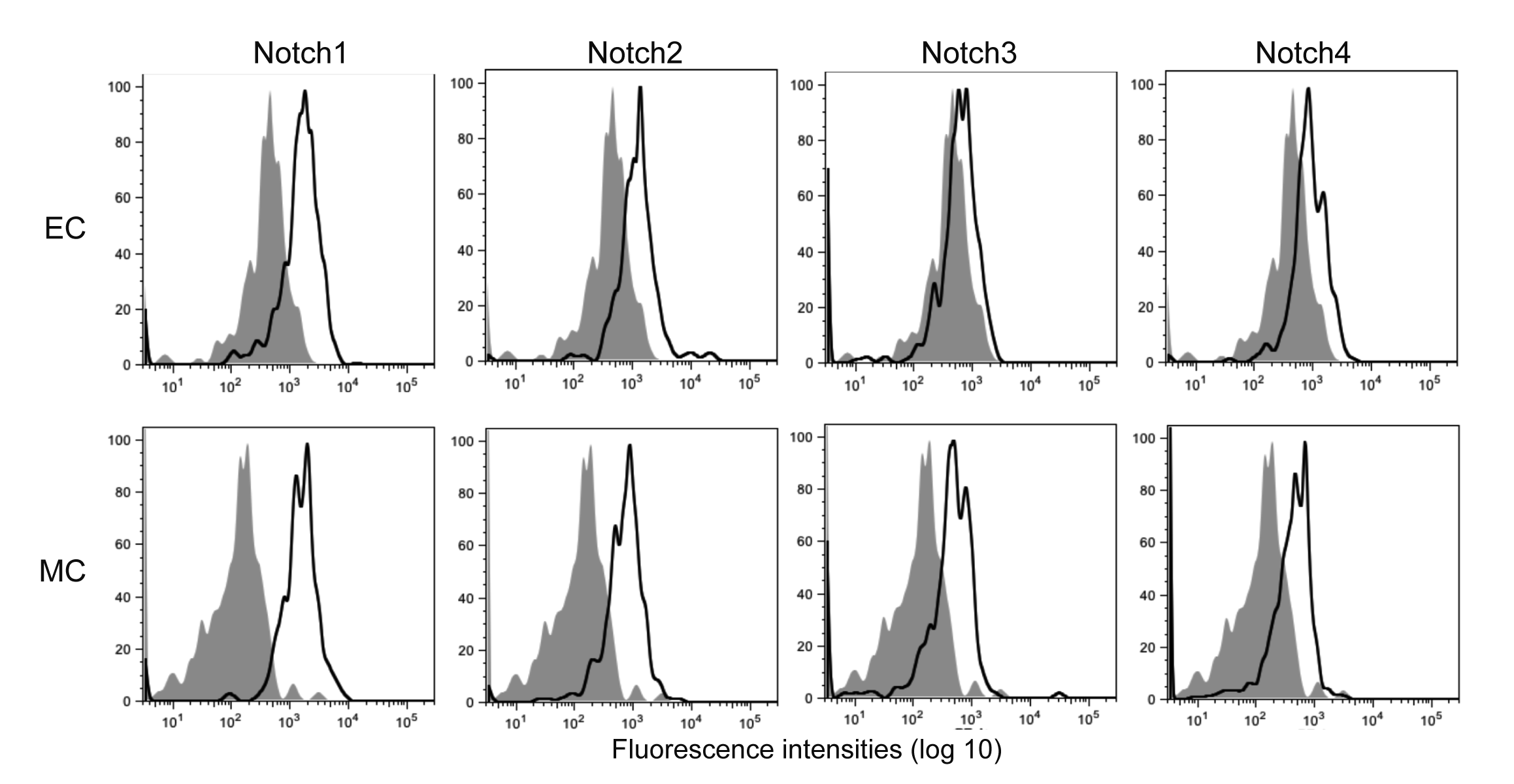

Supplement: S5 Fig — Cell surface expression of Notch receptors was analyzed for primary ECs and MCs from BM with FACS. Shaded areas indicate control staining. (TIF) [file pone.0134026.s005.tif]

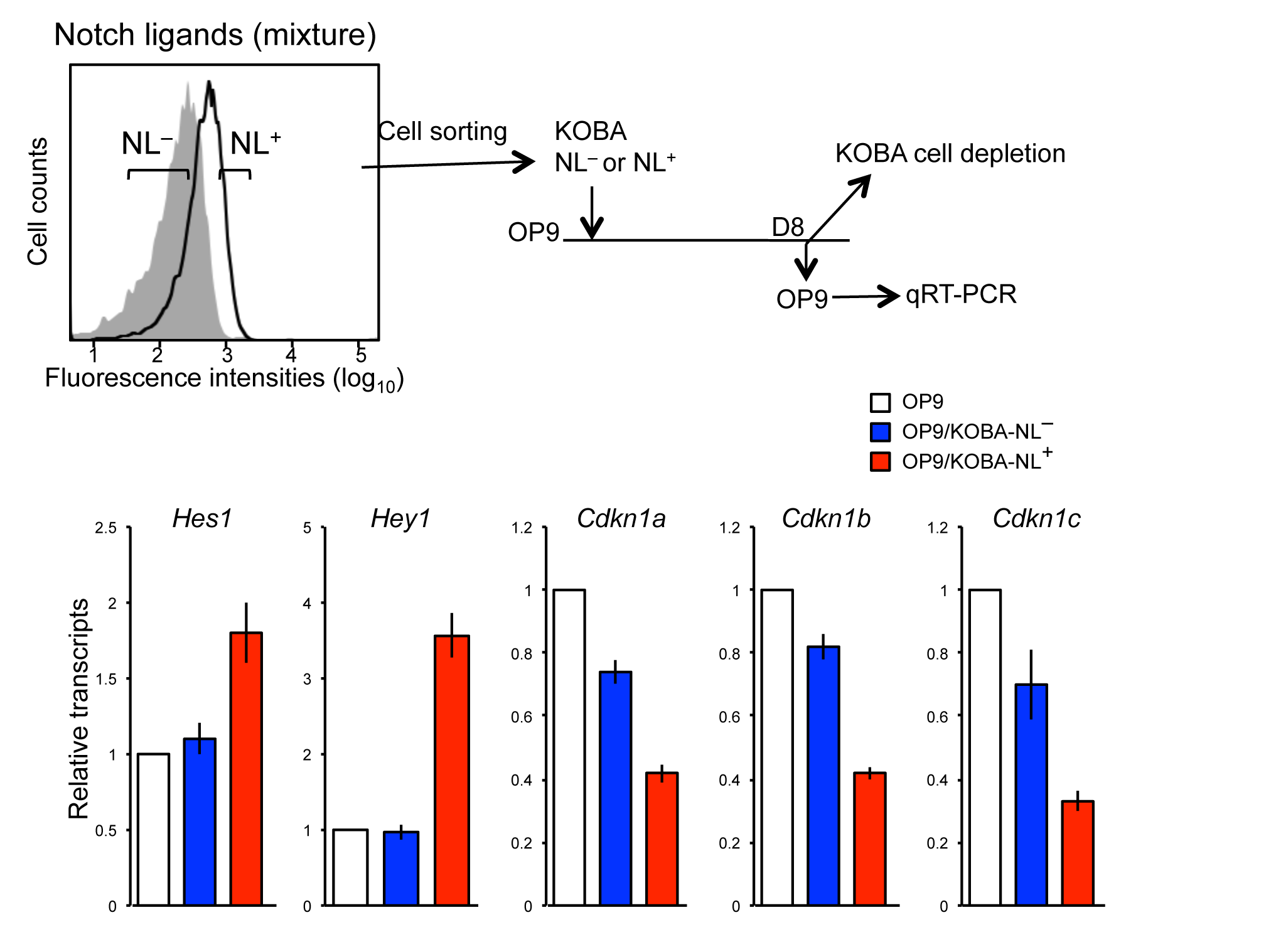

Supplement: S6 Fig — KOBA cells were stained with the mixture of anti-NL (Jagged1, Jagged2, Dll-1) antibodies, and the NL+ and NL− fractions were sorted as indicated with FACS AriaIII. Each fraction was cultured in the presence of OP9 cells for 8 days, OP9 cells were recovered after depleting CD45+ KOBA cells with AutoMax, and the expression of indicated genes were determined by quantitative RT-PCR. The means and SEs of triplicate determination are shown. (TIF) [file pone.0134026.s006.tif]

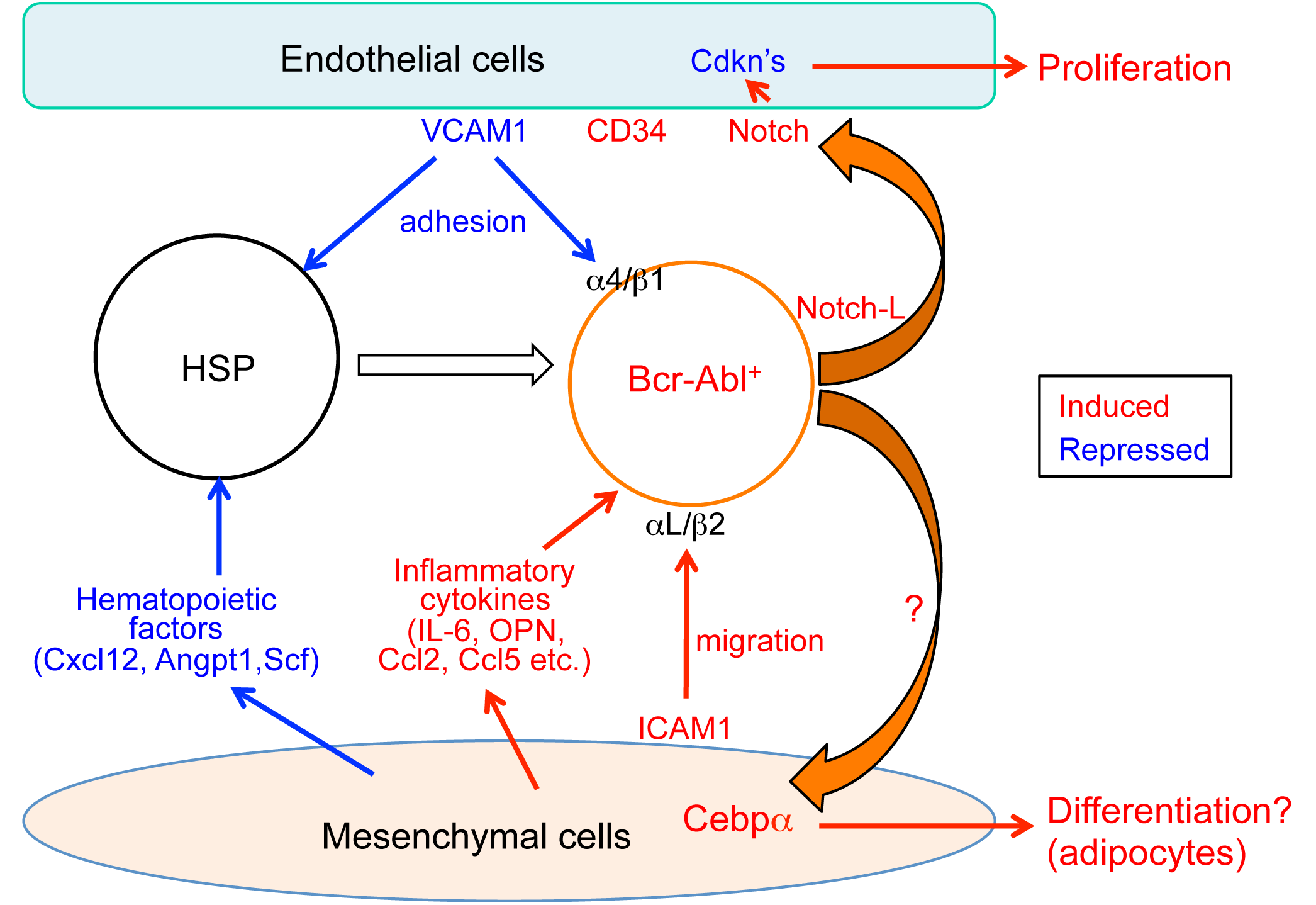

Supplement: S7 Fig — Bcr-Abl+ leukemia cells directly activate Notch signal, leading to the repression of Cdk inhibitor genes in ECs and neovasculogenesis. It is possible that the increase in ECs also involves the transdifferentiation from MSCs associated with CD34 expression. Notch activation in MCs causes increased ICAM1 expression, promoting the leukemia cell migration. Also, Bcr-Abl+ leukemia cells repress the hematopoietic genes but remarkably enhance the expression of diverse proinflammatory genes in MCs. The effects are Notch-independent and may involve the differentiation promotion to adipocytes. Such a drastic change in the cytokine milieu may favor the expansion of leukemia cells at the cost of normal hematopoiesis in the BM. HSPC; hematopoietic stem/progenitor cell. (TIF) [file pone.0134026.s007.tif]
