# Supplementary material for: Modification of Gene Expression, Proliferation, and Function of OP9 Stroma Cells by Bcr-Abl-Expressing Leukemia Cells
Source: PLoS One. 2015 Jul 28;10(7):e0134026. doi: 10.1371/journal.pone.0134026 (PMC4517910; doi:10.1371/journal.pone.0134026)
Supplement: S1 Table — (PDF) [file pone.0134026.s008.pdf]

Table S1. Altered gene expression in OP9 cells by the coculture with KOBA cells

Genes overexpressed in OP9/L cells

Genes	Ratio (log2)	Genes	Ratio (log2)	Genes	Ratio (log2)
Fosb	6.20	Bhlhe40	2.40	Ntrk3	1.72
Ubd	5.73	Junb	2.40	Btg1	1.72
Fos	5.63	Synm	2.38	Cx3cl1	1.71
Cd74	5.31	Tgtp2	2.36	Cxcr7	1.71
C3	5.24	Icam1	2.36	H2-T23	1.71
Gdpd2	5.20	Ier3	2.29	Map3k8	1.70
Ccl5	5.16	Sphk1	2.26	H2-Q2	1.70
Cxcl1	5.06	Tifa	2.22	Enpp3	1.69
Zfp36	5.00	Socs3	2.21	C1ra	1.69
Mmp13	4.94	Apoe	2.16	Cnnm4	1.67
Cfb	4.94	Arid5a	2.08	Irf1	1.67
Lcn2	4.71	Egr4	2.08	H2-M2	1.66
Ccl2	4.54	Nrg1	2.08	P2rx4	1.66
Nrp2	4.30	Cp	2.08	Pcdhb7	1.65
C4b	4.22	Malat1	2.08	Speer4b	1.65
Serpina3g	4.13	Bmper	2.05	H2-D1	1.64
Mt1	3.97	Tnxb	2.04	Dclk1	1.64
Enpp2	3.83	Postn	2.00	Adamts9	1.63
Chadl	3.62	Pim1	2.00	Ankrd37	1.63
Crip1	3.61	Nat8	1.99	Adamts9	1.63
Jun	3.44	Ppp1r15a	1.99	Slit2	1.61
Mt2	3.44	Eno2	1.97	Zc3h12a	1.61
Atf3	3.36	Pip5k1a	1.97	Ptgir	1.61
Zmynd15	3.34	Tnfsf11	1.97	Ank3	1.60
Kng1	3.21	Slco1a5	1.95	Ly6e	1.59
H2-K1	3.15	Ninl	1.93	Car15	1.59
Lrg1	3.10	Nr4a1	1.91	Hist1h1c	1.59
Nfkbiz	3.09	Socs1	1.90	Rarres2	1.59
Ier2	3.02	Sparcl1	1.88	Bcl3	1.58
Ramp2	3.01	Tgm2	1.88	Slc12a5	1.57
Cxcl5	2.86	H2-K2	1.86	Plod2	1.57
Tlr2	2.85	H2-Bl	1.85	Med12	1.57
Chrdl1	2.85	Spns2	1.84	Rgs11	1.57
Ak4	2.83	Slc23a3	1.84	Ism1	1.56
Ccl7	2.78	Igsf3	1.83	Arrdc4	1.55
Sfrp4	2.72	Agt	1.82	Lifr	1.55
Ptgs2	2.72	Col3a1	1.79	Hes1	1.55
Apod	2.71	Gpr146	1.79	Dpysl3	1.54
Dnahc6	2.71	Dusp1	1.78	Gcm1	1.54
Vpreb1	2.66	Nfkbia	1.78	Cygb	1.52
Apln	2.63	Cfh	1.78	Ablim1	1.52
Dtx4	2.61	Ltbp2	1.77	Fgd6	1.51
Sfrp2	2.60	Kif21b	1.77	Il6	1.47
Nfkbie	2.53	Ninl	1.77	Col20a1	1.34
Gfra4	2.53	H2-Q7	1.76	Cdh11	1.27
Btg2	2.50	C1rb	1.75	Pdgfra	1.22
C1s	2.49	Egr2	1.74	Cdh26	1.05
Tgfbi	2.43	Cxadr	1.73	Vegfa	0.66
Frat2	2.40	H2-Q6	1.73		
Esm1	2.40	Scara5	1.72		

Genes underexpressed in OP9/L cells

Genes	Ratio (log2)		Genes	Ratio (log2)		Genes	Ratio (log2)
Cdkn2b	-0.96		Eif2s3y	-1.72		Cdkn1c	-2.18
Cdkn1a	-1.06		Ddx3y	-1.73		Cryab	-2.18
Cdkn1b	-1.16		Svop	-1.73		Cd109	-2.18
Cdkn3	-1.30		Sorbs2	-1.73		Mgp	-2.19
Pde1b	-1.50		Slitrk5	-1.77		Gcnt1	-2.22
Cd44	-1.50		S100a4	-1.77		Tbc1d2	-2.22
Tram1l1	-1.51		Dlk1	-1.78		Mfap5	-2.23
Filip1l	-1.52		Tes	-1.78		Cd24a	-2.28
Lmcd1	-1.52		H19	-1.83		Chac1	-2.30
Gale	-1.52		Scx	-1.84		Klhdc9	-2.36
Hspb1	-1.53		Itgad	-1.86		Bcat1	-2.41
Cd97	-1.53		Myom1	-1.86		Anxa8	-2.43
Ass1	-1.56		Fgd3	-1.88		Inhba	-2.53
Rassf7	-1.56		Sncg	-1.92		Pdlim5	-2.68
Hps1	-1.60		Scrn1	-1.95		Prl2c5	-2.93
Atp9a	-1.60		Lgr5	-1.97		Siglecg	-2.99
Rem1	-1.60		Lbh	-1.99		Ifi27l2a	-3.00
Sdr39u1	-1.62		Des	-2.00		Prl2c5	-3.01
Smarca1	-1.62		Npas4	-2.03		Plb1	-3.52
Dysf	-1.63		Cryab	-2.04		Syt10	-3.62
Trim16	-1.63		Aif1l	-2.05		Aldh1a7	-3.84
Mogat2	-1.64		S100b	-2.08		Tmem184a	-4.40
Ndrg4	-1.65		Mcf2l	-2.10			
Col18a1	-1.66		Cnn1	-2.11			
Idi1	-1.68		Mirg	-2.14			
Comp	-1.70		Sykb	-2.17			
