# Supplementary material for: Modification of Gene Expression, Proliferation, and Function of OP9 Stroma Cells by Bcr-Abl-Expressing Leukemia Cells
Source: PLoS One. 2015 Jul 28;10(7):e0134026. doi: 10.1371/journal.pone.0134026 (PMC4517910; doi:10.1371/journal.pone.0134026)
Supplement: S2 Table — (PDF) [file pone.0134026.s009.pdf]

Table S2. List of the primer pairs

Genes	sense	anti-sense
Ccl2	catccacgtgttggctca	gatcatcttgctggtgaatgagt
Ccl5	tgcagaggactctgagacagc	gagtggtgtccgagccata
Ccl7	ttctgtgcctgctgctcata	ttgacatagcagcatgtggat
Cxcl1	gactccagccacactccaac	tgacagcgcagctcattg
Cxcl5	tagagccccaatctccacac	gagctggaggctcattgtg
C3	accttacctcggcaagtttct	ttgtagagctgctggtcagg
C4	tctcacaaacccctcgacat	agcatcctggaacacctgaa
Spp1	cccggtgaaagtgactgatt	ttcttcagaggacacagcattc
Icam1	cccacgctacctctgctc	gatggatacctgagcatcacc
CD44	ctccttctttatccggagcac	tggctttttgagtgcacagt
Itga3	tcaacatggagaacaagacca	ccaaccacagctcaatctc
Itgb3	gtgggagggcagtcctcta	caggatatcaggacccttgg
Cdh13	cactcaccaaaattcaccaaga	ttgacaatgacccccacag
Scf	tcaacattaggtcccgagaaa	actgctactgctgtcattcctaag
Cxcl12	ccaaactgtgcccttcaga	atttcgggtcaatgcacact
Angp1	ggaagatggaagcctggat-	accagagggattcccaaaac
Cdkn1a	tccacagcgatatccagaca	ggacatcaccaggattggac
Cdkn1b	gttagcggagcagtgtcca	tctgttctgttggccctttt
Cdkn1c	caggacgagaatcaagagca	gcttggcgaagaagtcgt
Cdkn3	gatgaagaacagactccaattcaa	aacctggaagagcacataaacc
Hey1	catgaagagagctcacccaga	cgccgaactcaagtttcc
Hey2	gtggggagcgagaacaatta	gttgtcggtgaattggacct
Hes1	tgccagctgatataatggagaa	ccatgataggctttgatgacttt
